# Supplementary material for: Whole-genome analysis of Nigerian patients with breast cancer reveals ethnic-driven somatic evolution and distinct genomic subtypes
Source: Nat Commun. 2021 Nov 26;12:6946. doi: 10.1038/s41467-021-27079-w (PMC8626467; doi:10.1038/s41467-021-27079-w)
Supplement: Supplementary file 1 — Supplementary Information [file 41467_2021_27079_MOESM1_ESM.pdf]

Supplementary Information

Whole-genome Analysis of Nigerian Patients with Breast Cancer Reveals Ethnic-driven Somatic Evolution and Distinct Genomic Subtypes

Ansari-Pour N & Zheng Y *et al.*

Supplementary Tables

Supplementary Table 1: Characteristics of study participants.

Group	Nigerian Black (Nigerian)	Black TCGA (Black)	White TCGA (White)
N	97	30	46
Age at diagnosis, mean (SD)	50.8 (12.8)	57.0 (14.1)	60.2 (13.2)
Subtype			
HER2+	40 (41%)	0 (0%)	15 (33%)
HR+/HER2-	16 (16%)	10 (33%)	13 (28%)
HR-/HER2-	40 (41%)	19 (63%)	17 (37%)
NA	1 (1%)	1 (3%)	1 (2%)
Ploidy, median (IQR)	3.24 (2.82, 3.77)	3.01 (2.06, 3.77)	2.98 (2.05, 3.44)
Tumor purity, median (IQR)	0.44 (0.28, 0.63)	0.54 (0.43, 0.69)	0.66 (0.55, 0.77)
Histology			
Ductal	86 (89%)	24 (80%)	42 (91%)
Lobular	2 (2%)	2 (7%)	2 (4%)
Other	9 (9%)	4 (13%)	2 (4%)
Proportion of African ancestry, mean (SD)	1.00 (0.00)	0.79 (0.13)	0.00 (0.01)

HR, hormone receptor; HER2, human epidermal growth factor receptor 2; IQR, interquartile range; SD, standard deviation

Supplementary Table 2: Selected risk factors for patients in the Nigerian study.

Factor	Value
Age at diagnosis	
<35	6 (6%)
35-44	28 (29%)
45-54	29 (30%)
55-64	18 (19%)
>=65	16 (16%)
mean (SD)	50.8 (12.8)
Age at menarche, mean (SD)	14.9 (1.9)
Num. of live births	
0	5 (5%)
1-2	29 (32%)
3-4	31 (34%)
5+	27 (29%)
mean (SD)	3.3 (2.0)
Months of total lactation, mean (SD)	50.3 (36.9)
Age at first live birth, mean (SD)	25.0 (5.2)
Family history of breast cancer	
No	92 (100%)
Benign breast diseases	
Yes	5 (5%)
No	87 (95%)
Oral contraceptives	
Yes	19 (20%)
No	74 (80%)
Alcohol consumption	
Yes	5 (5%)
No	88 (95%)
Height in cm, mean (SD)	163.0 (7.6)
Body mass index in Kg/m2	
<25	39 (44%)
25-29.9	24 (27%)
30+	25 (28%)
mean (SD)	26.4 (5.6)

SD, standard deviation

Supplementary Table 3: Characteristics of the somatic mutational drivers detected in breast tumors.

Driver	Detection Method	Mutational Pattern	Cancer Gene Status
ADAM19	cDriver	Neither	Previously unreported
BCLAF1	MutSigCV	TSG	COSMIC
BYSL	cDriver	TSG	Previously unreported
CDH1	cDriver/MutSigCV	TSG	COSMIC
F5	cDriver	TSG	Previously unreported
GATA3	cDriver/MutSigCV	TSG	COSMIC
LAMB3	cDriver	Neither	Previously unreported
MAP3K1	cDriver/MutSigCV	TSG	COSMIC
PIK3CA	cDriver/MutSigCV	ONC	COSMIC
PTEN	cDriver/MutSigCV	TSG	COSMIC
RB1	cDriver	TSG	COSMIC
TDRD15	cDriver	TSG	Previously unreported
TP53	cDriver/MutSigCV	TSG/ONC	COSMIC

TSG, tumor suppressor gene; ONC, oncogene; COSMIC, Catalogue of Somatic Mutations in Cancer

Supplementary Figures

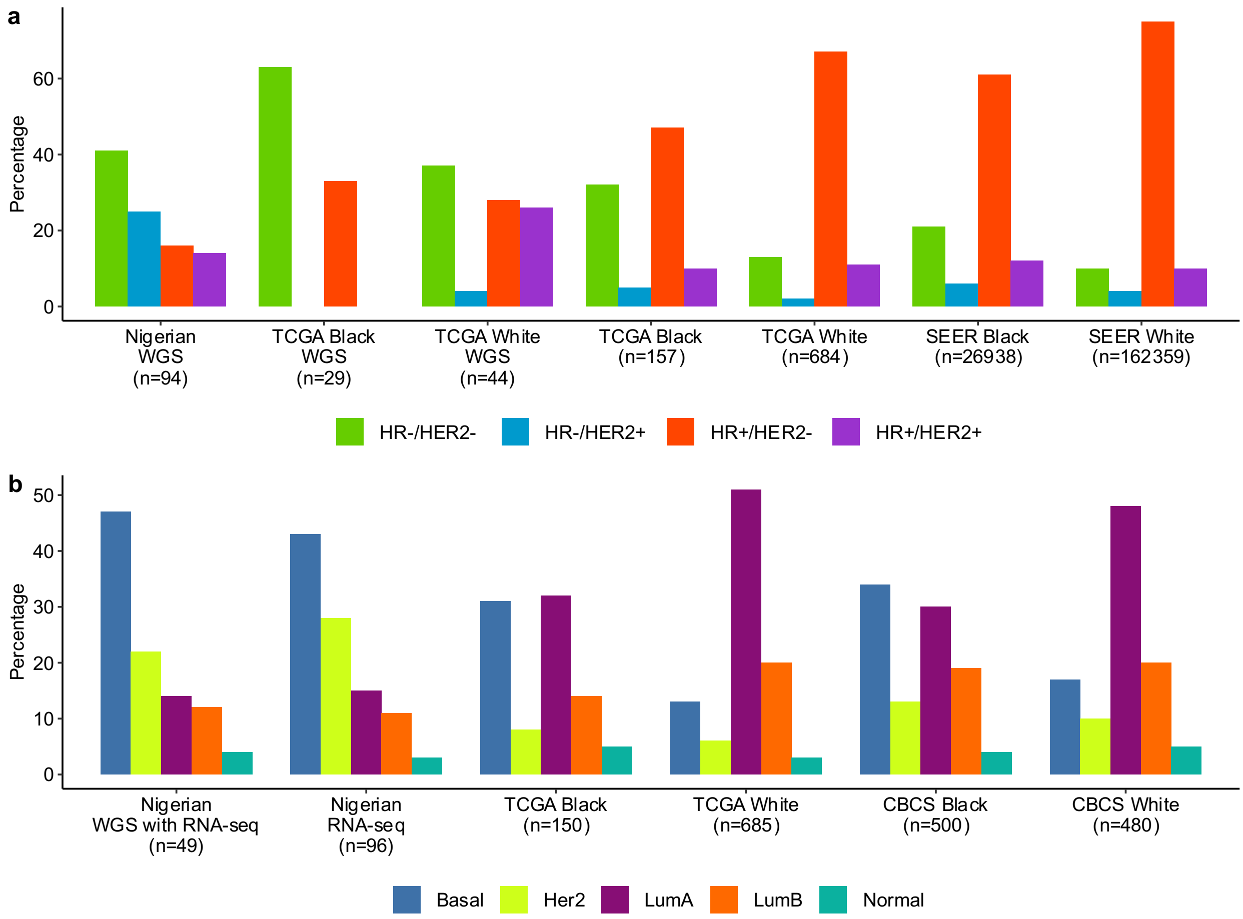

Supplementary Fig. 1 Proportion of immunohistochemistry (IHC) and PAM50 subtypes in Nigerians and cohorts in TCGA (The Cancer Genome Atlas), SEER (Surveillance, Epidemiology, and End Results Program) and CBCS (Carolina Breast Cancer Study). **a** IHC subtype distribution across populations, data sources: 'Nigerian WGS', 'TCGA Black WGS', and 'TCGA White WGS' are from Pitt JJ, *et al.* 2018 (PMID: 30327465), the larger TCGA data ('TCGA Black' and 'TCGA White') are from TCGA PanCancer Atlas and Firehose Legacy at cBioPortal (https://www.cbioportal.org/), 'SEER Black' and 'SEER White' are from Kong X *et al.* 2020 (PMID: 33074325). **b** PAM50 subtype distribution across populations, data sources: 'Nigerian WGS with RNA-seq' and 'Nigerian RNA-seq' are from the current study, 'TCGA Black' and 'TCGA White') are from TCGA PanCancer Atlas and Firehose Legacy at cBioPortal (https://www.cbioportal.org/), 'CBCS Black' and 'CBCS White' are from Troester MA. *et al.* 2017 (PMID: 28859290). Source data are provided as a Source Data file. Sample size of each group is indicated in the figure. WGS, whole-genome sequencing; HR, hormone receptor; HER2, human epidermal growth factor receptor 2; LumA/B, luminal A/B.

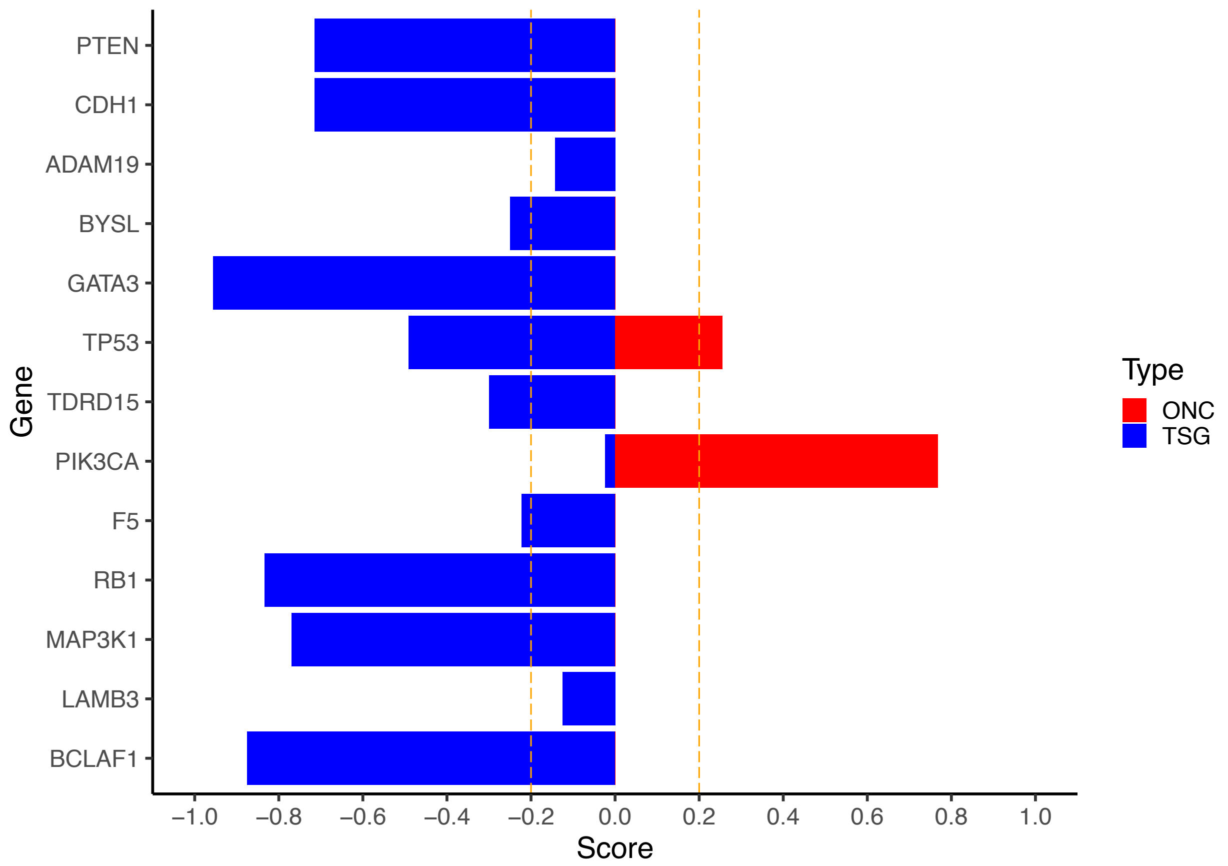

Supplementary Fig. 2 Oncogene and tumor suppressor gene categorization scores for detected mutational drivers. The dashed orange vertical lines represent the threshold scores according to the 20/20 rule. TSG, tumor suppressor gene; ONC, oncogene.

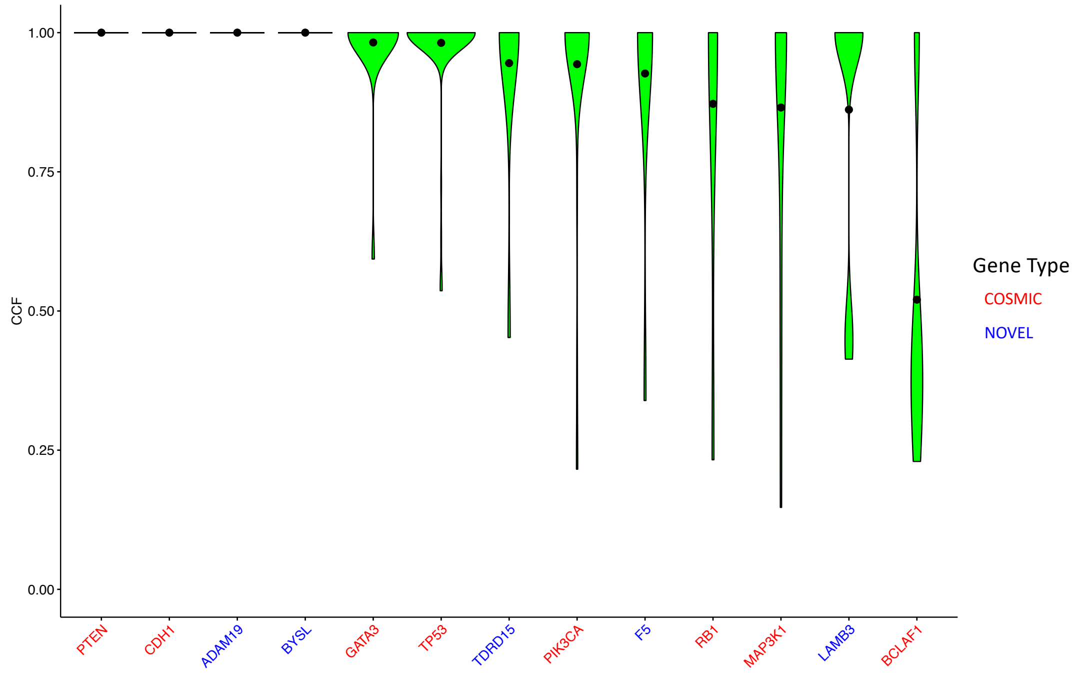

Supplementary Fig. 3 Cancer cell fraction (CCF) distribution of mutations in detected mutational drivers.

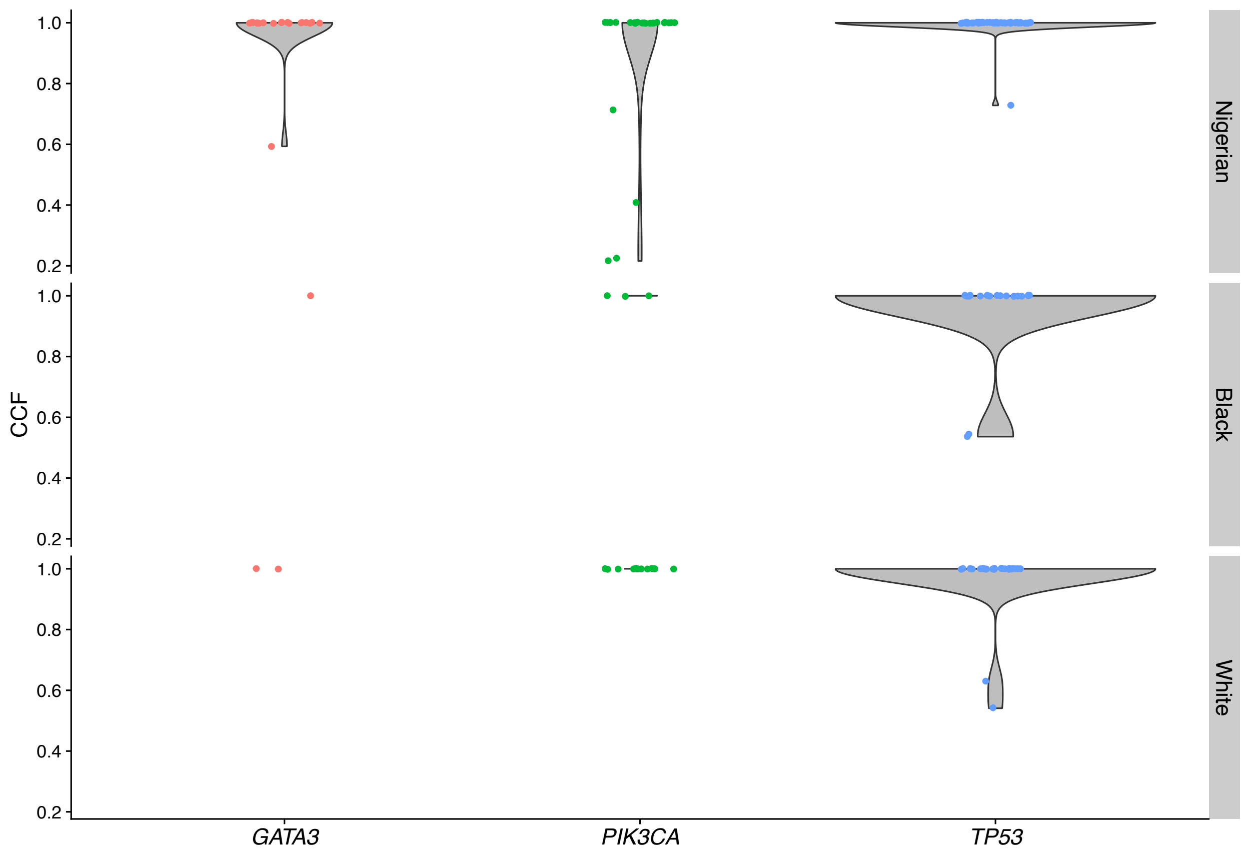

Supplementary Fig. 4 Cancer cell fraction (CCF) distribution of the top mutated genes (>10%) in all three groups.

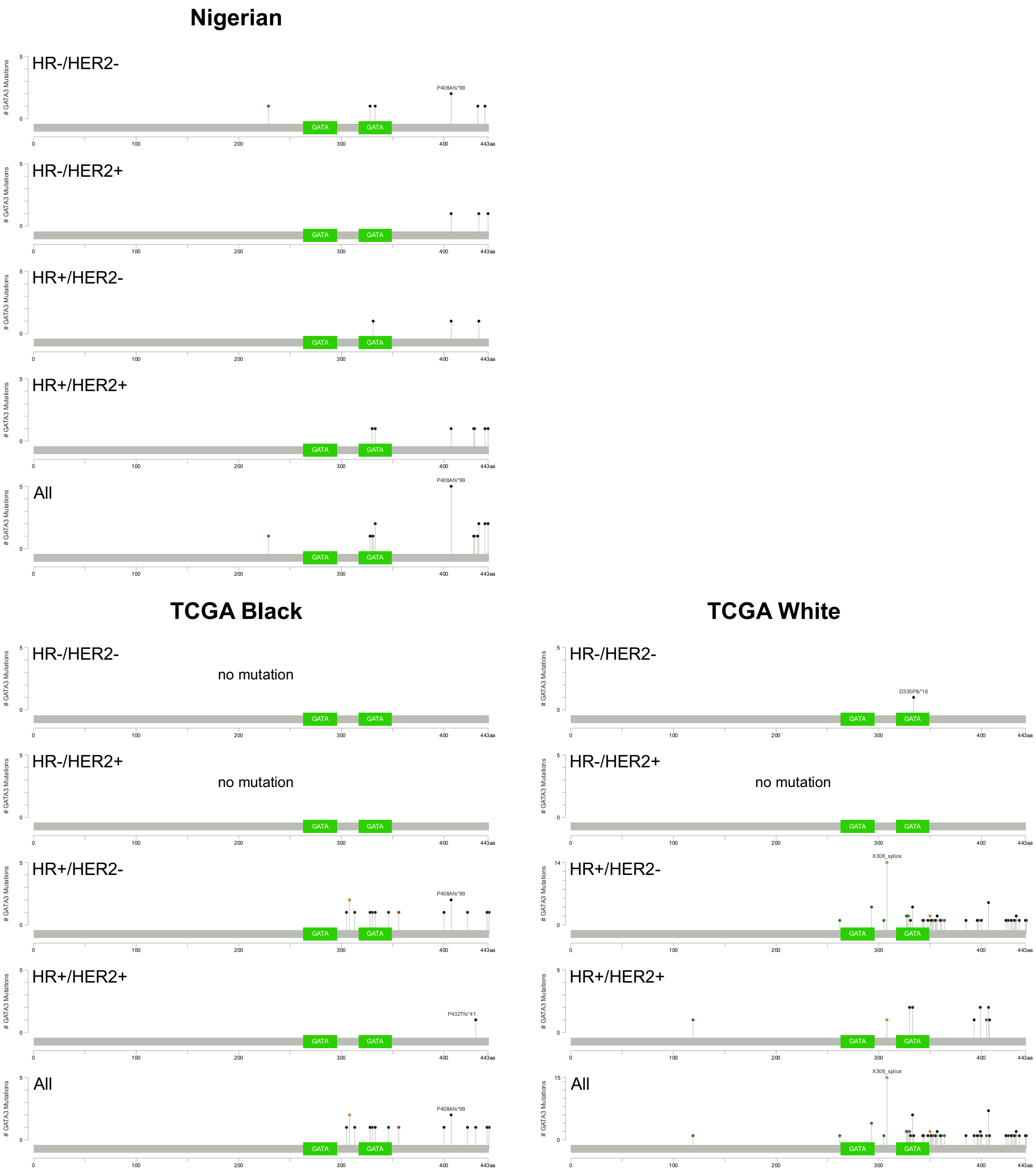
Nigerian
TCGA Black
TCGA White
HR-/HER2-
HR-/HER2+
HR+/HER2-
HR+/HER2+
All
no mutation
GATA3 Mutations
GATA
P408Afs*99
P432Tfs*41
D335Pfs*16
X308_splice
0
100
200
300
400
443aa

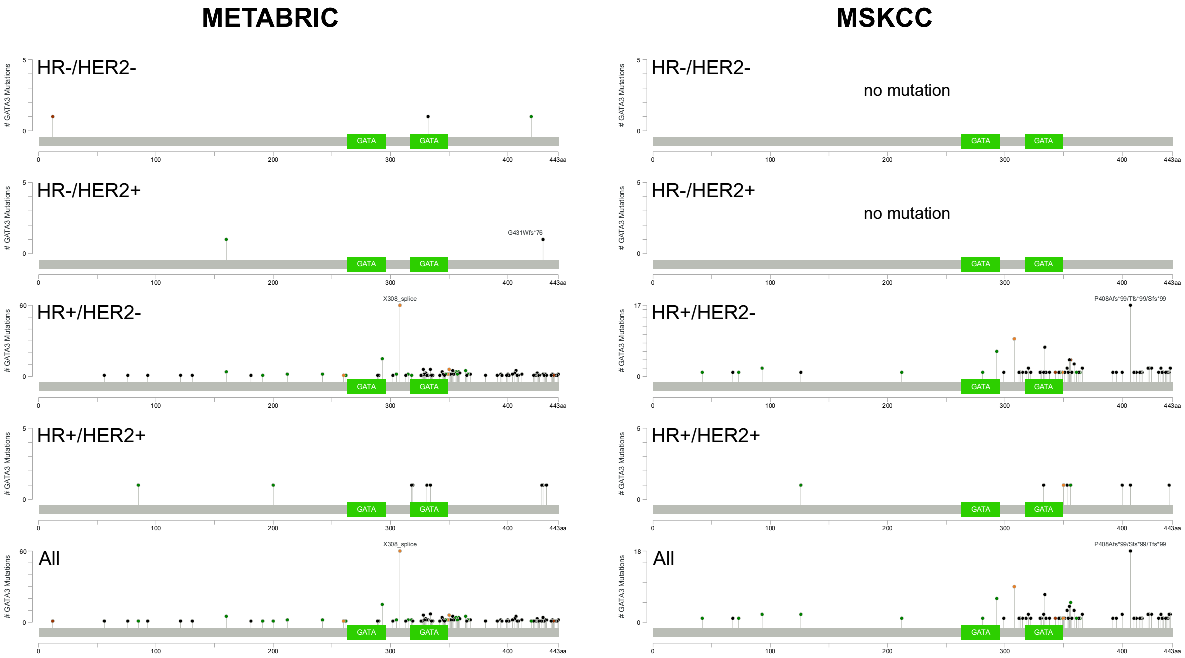

Supplementary Fig. 5 Lollipop plot illustrates the *GATA3* mutations across immunohistochemistry (IHC) subtypes in different studies. Data sources: Nigerian whole-genome sequencing are from the present study; TCGA (The Cancer Genome Atlas) whole-exome sequencing, METABRIC (Molecular Taxonomy of Breast Cancer International Consortium) targeted sequencing, and MSKCC (Memorial Sloan Kettering Cancer Center) targeted sequencing are from cBioPortal (https://www.cbioportal.org/). HR, hormone receptor; HER2, human epidermal growth factor receptor 2.

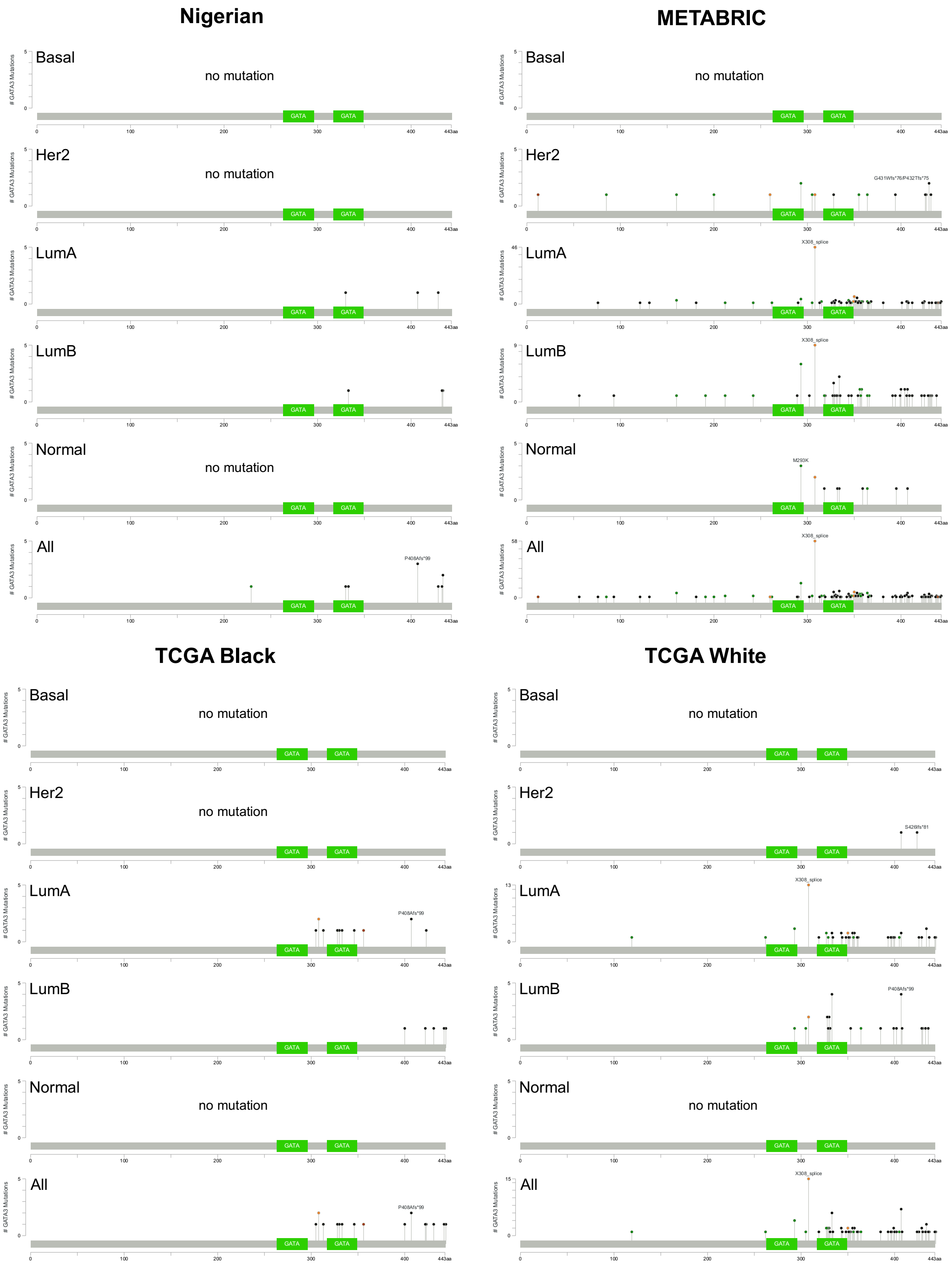

Nigerian
METABRIC
TCGA Black
TCGA White
Basal
Her2
LumA
LumB
Normal
All
no mutation
GATA3 Mutations
GATA
443aa
G431Wfs*76/P432Tfs*75
X308_splice
M293K
P408Afs*99
S426Ifs*81

Supplementary Fig. 6 Lollipop plot illustrates the *GATA3* mutations across PAM50 subtypes in different studies. Data sources: Nigerian whole-genome sequencing are from the present study; TCGA (The Cancer Genome Atlas) whole-exome sequencing, METABRIC (Molecular Taxonomy of Breast Cancer International Consortium) targeted sequencing are from cBioPortal (https://www.cbioportal.org/). Her2, human epidermal growth factor receptor 2; LumA/B, luminal A/B.

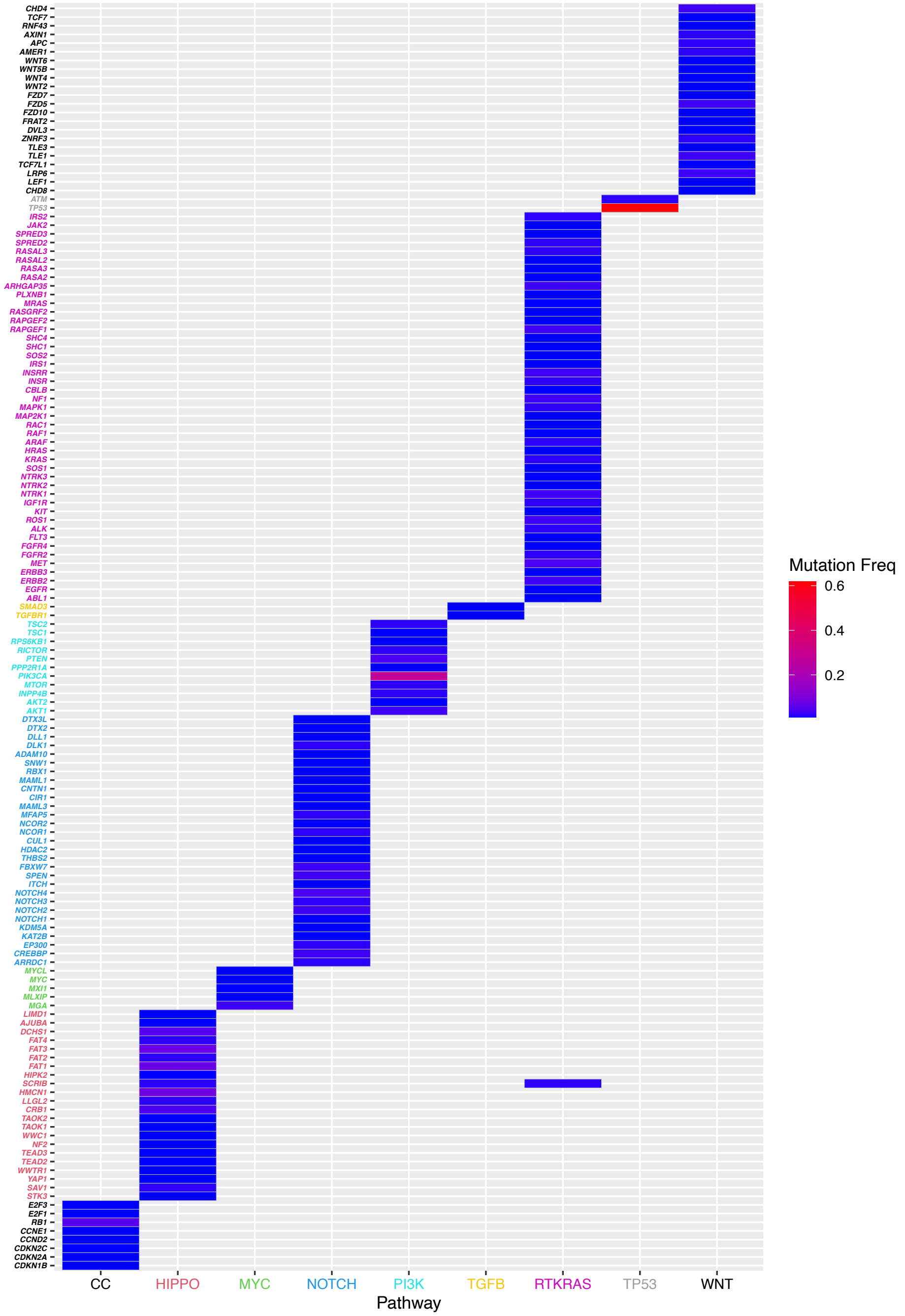

Supplementary Fig. 7 Heatmap of mutation frequency in genes associated with canonical cancer pathways in the Nigerian group. Genes are colored according to their primary associated canonical pathway.

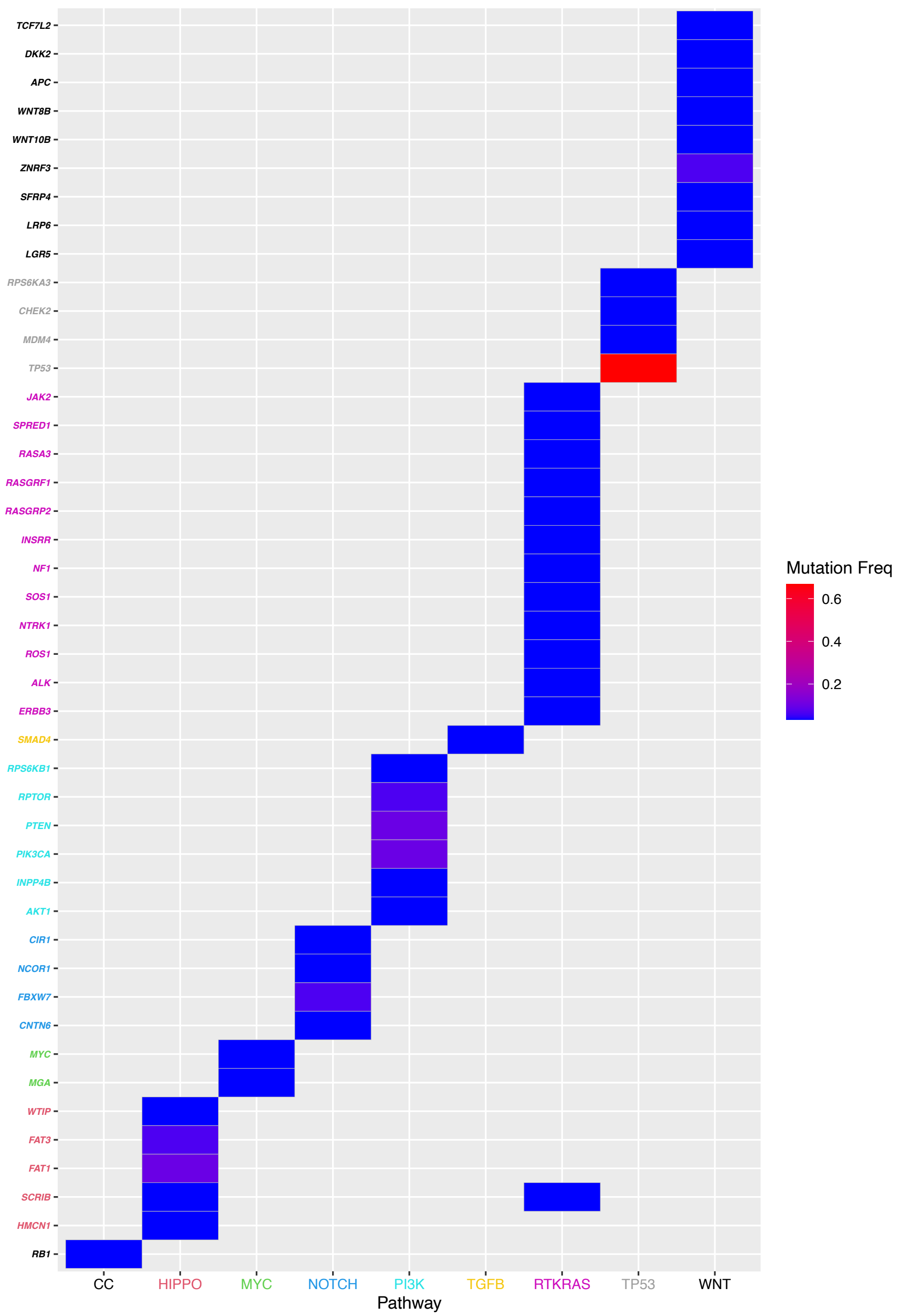

Supplementary Fig. 8 Heatmap of mutation frequency in genes associated with canonical cancer pathways in the Black group. Genes are colored according to their primary associated canonical pathway.

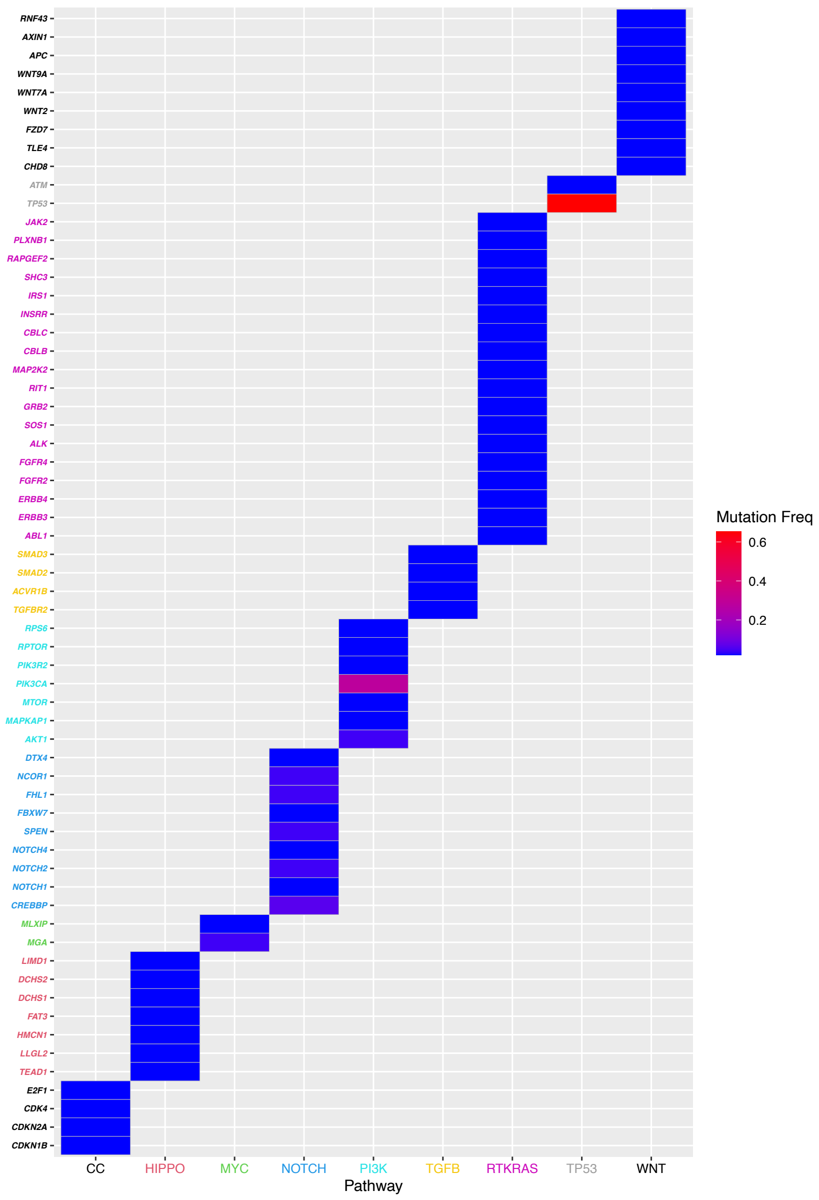

Supplementary Fig. 9 Heatmap of mutation frequency in genes associated with canonical cancer pathways in the White group. Genes are colored according to their primary associated canonical pathway.

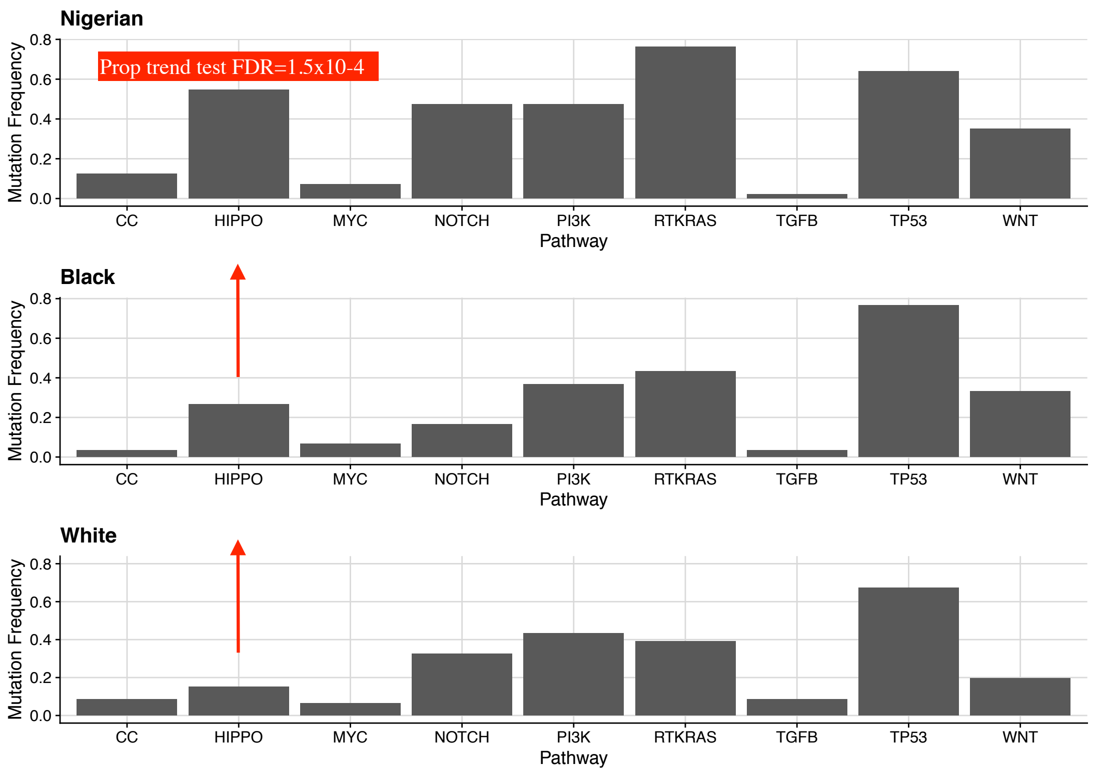

Supplementary Fig. 10 Comparison of combined mutation frequency in canonical cancer pathways in all three groups. Red arrows point out that the HIPPO pathway shows a strong positive cline in mutation frequency with increased African ancestry. Proportion trend test two-sided P value adjusted for multiple testing using FDR. Source data are provided as a Source Data file.

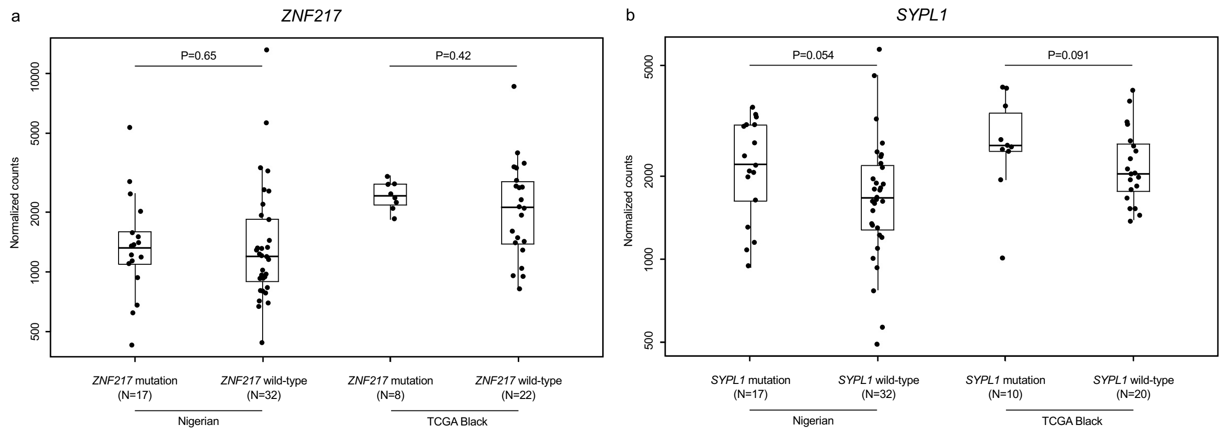

Supplementary Fig. 11 Gene expression among samples with versus without mutations in non-coding regions of **a** *ZNF217* and **b** *SYPL1*. All 49 Nigerian and 30 TCGA Black RNA-seq samples are biologically independent samples. Sample size of each comparison group is indicated in the figure. Reported counts are normalized based on the median of ratios method as employed by DESeq2, so as to account for sequencing depth and potential skewing of differential expression in each group. Each box represents the upper and lower quartiles of the data, and the median is depicted with a horizontal line. Upper and lower whiskers extend to the largest and smallest values within (1.5 × interquartile range), respectively. Differences by ancestry were not significant based on Wilcoxon rank sum testing. No significance was found as unadjusted two-sided P>0.05.

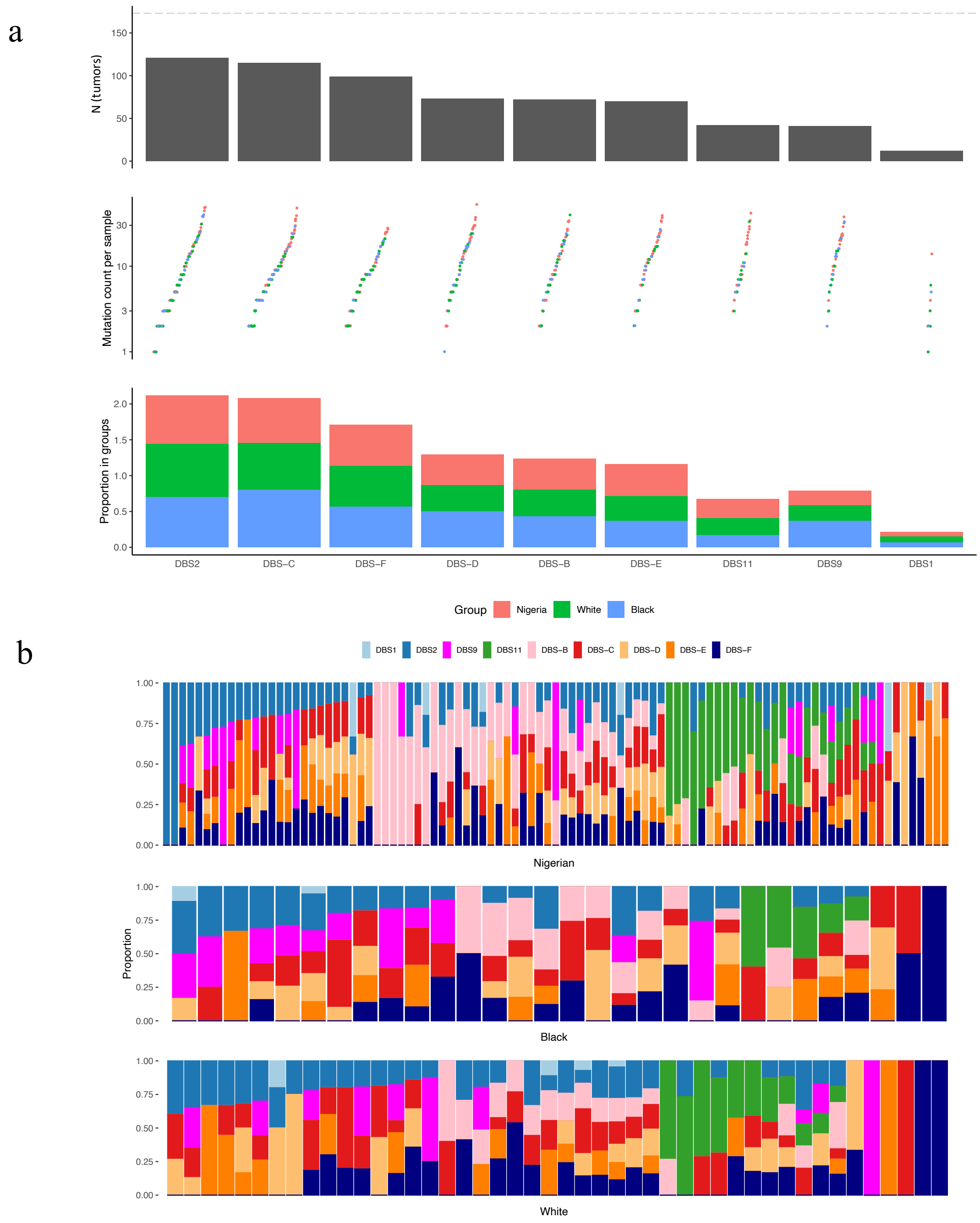

Supplementary Fig. 12 Double base substitution (DBS) signatures in all groups. **a** from top to bottom: number of tumors with DBS signatures across the entire dataset (dotted line represents total sample size, N=173) with signatures sorted left to right by descending frequency, number of mutations per sample (color representing groups) in respective signatures and proportion of samples carrying each signature in each group. **b** proportion of mutations assigned to each DBS signature across the three groups. Source data are provided as a Source Data file.

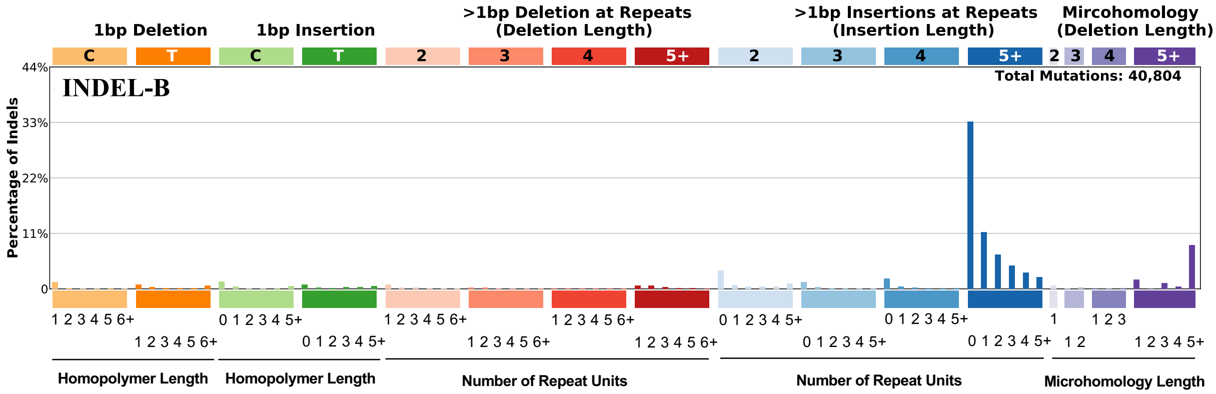

Supplementary Fig. 13 Mutational profile of the previously unreported INDEL-B signature. INDEL, insertion and deletion.

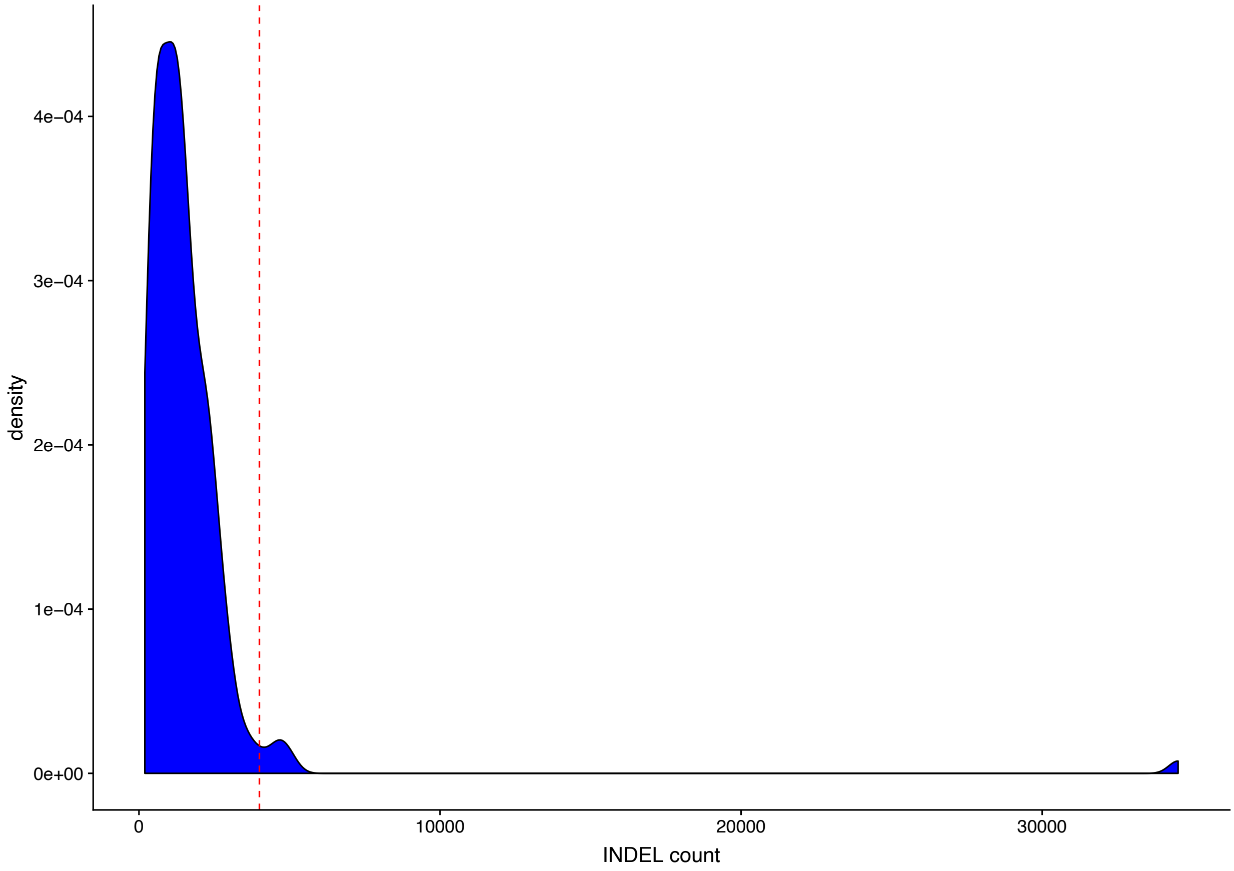

Supplementary Fig. 14 Bimodal distribution of INDEL variant count in Nigerian breast cancer tumors. INDEL, indel and deletion.

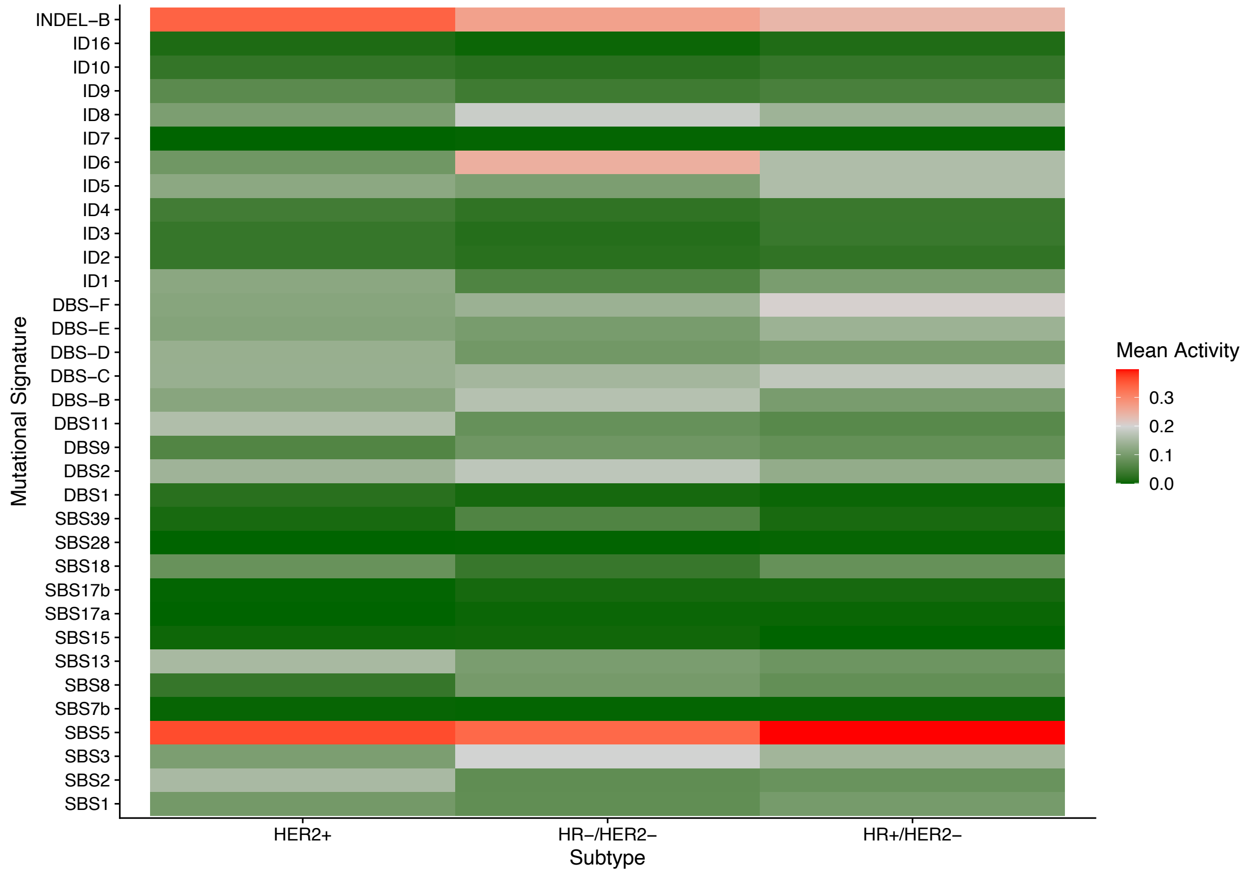

Supplementary Fig. 15 Mean activity of SBS, DBS and INDEL mutational signatures across clinical subtypes in breast cancer tumors. SBS, single base substitution; DBS, double base substitution; INDEL, indel and deletion.

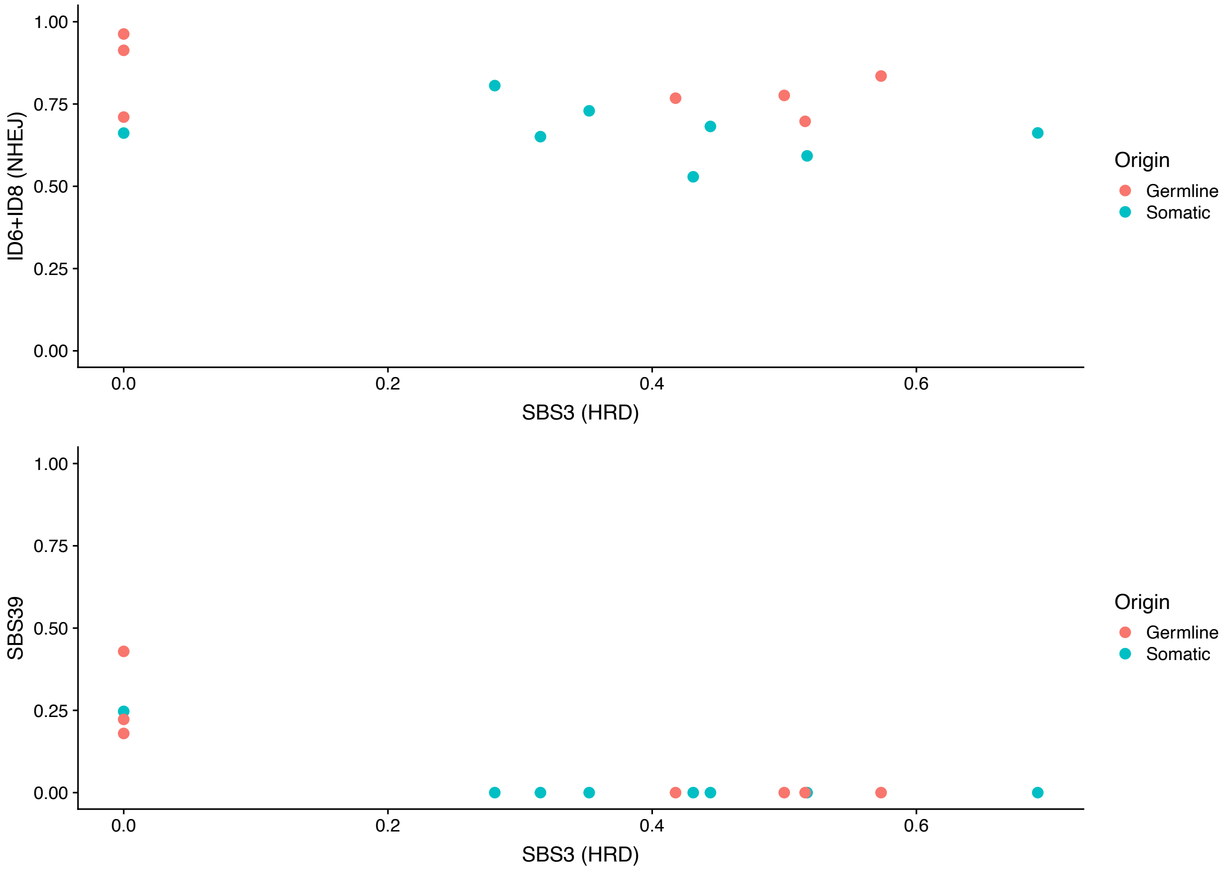

Supplementary Fig. 16 Activity levels of NHEJ, HRD and SBS39 signatures in *BRCA*-positive tumors. Flat signatures SBS39 and SBS3 (HRD) are observed mutually exclusively. NHEJ, non-homologous end joining; HRD, homologous recombination deficiency; SBS, single base substitution.

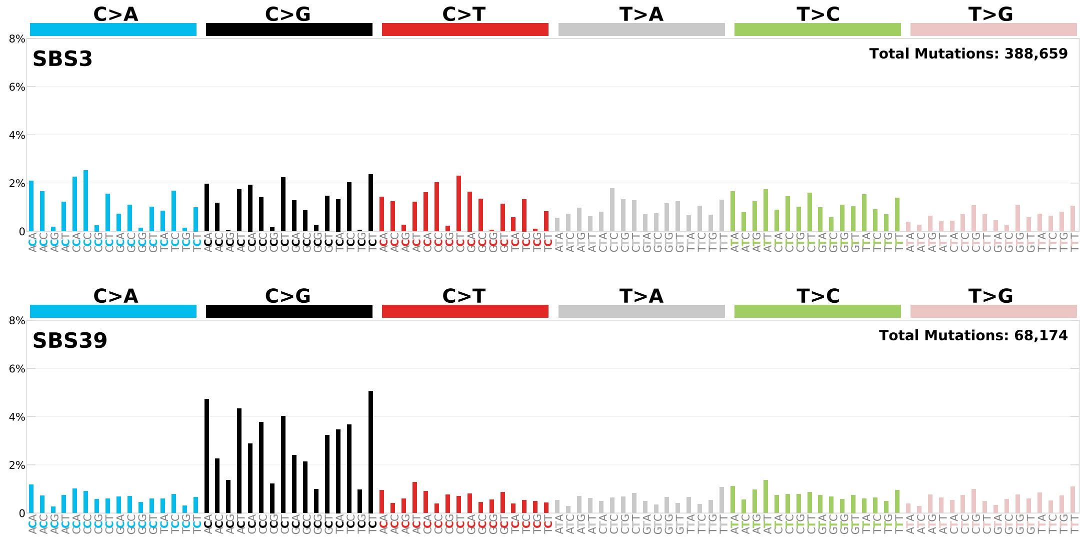

Supplementary Fig. 17 Mutational profile of signatures SBS3 and SBS39. SBS, single base substitution.

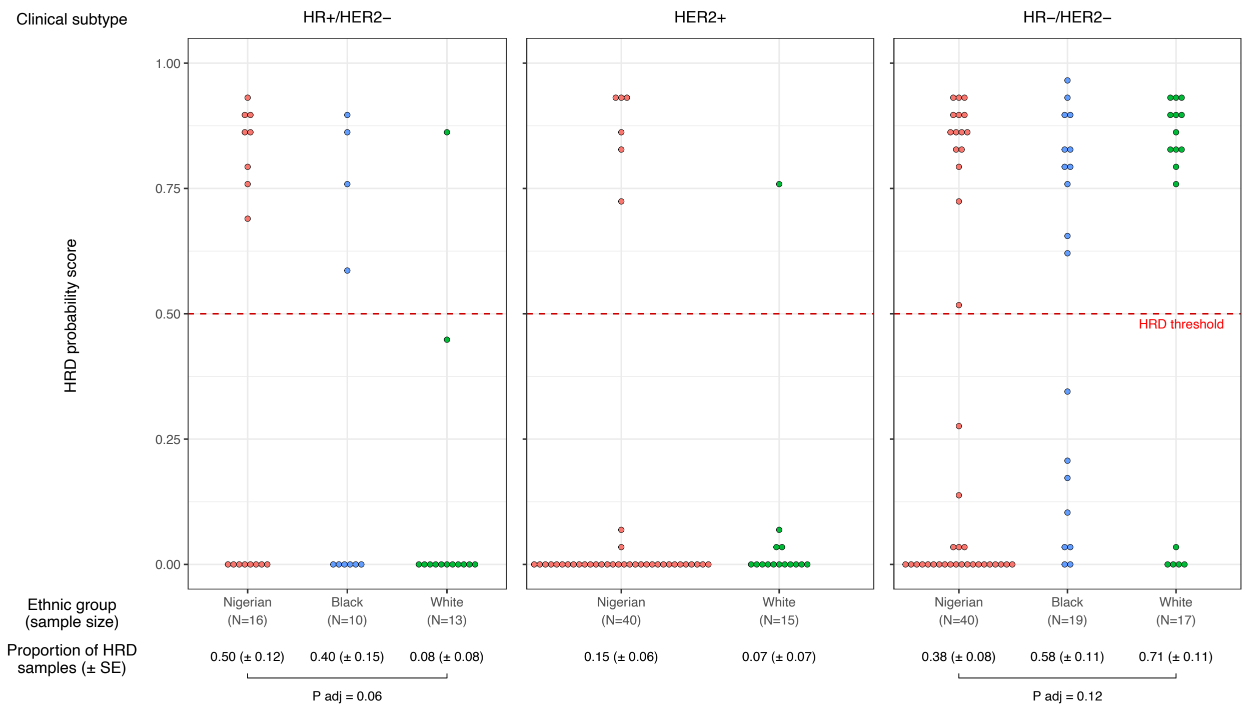

Supplementary Fig. 18 Analysis of CHORD results across clinical subtypes and ethnic groups. Homologous recombination deficiency (HRD) probability score for each sample is presented as a dot. Samples are considered homologous repair-deficient if P(HRD)>0.5. Proportion of samples predicted to have HRD across all ethnic groups and subtypes. All Nigerian, Black and White samples are biologically independent samples. Sample size of each comparison group is indicated in the figure. A two-tailed Fisher exact test was performed on each pair of ethnic groups within each clinical subtype. After adjusting for multiple testing with the Holm-Bonferroni correction, no pairwise comparison reached statistical significance ($P_{adj}<0.05$). SE, standard error of each proportion.

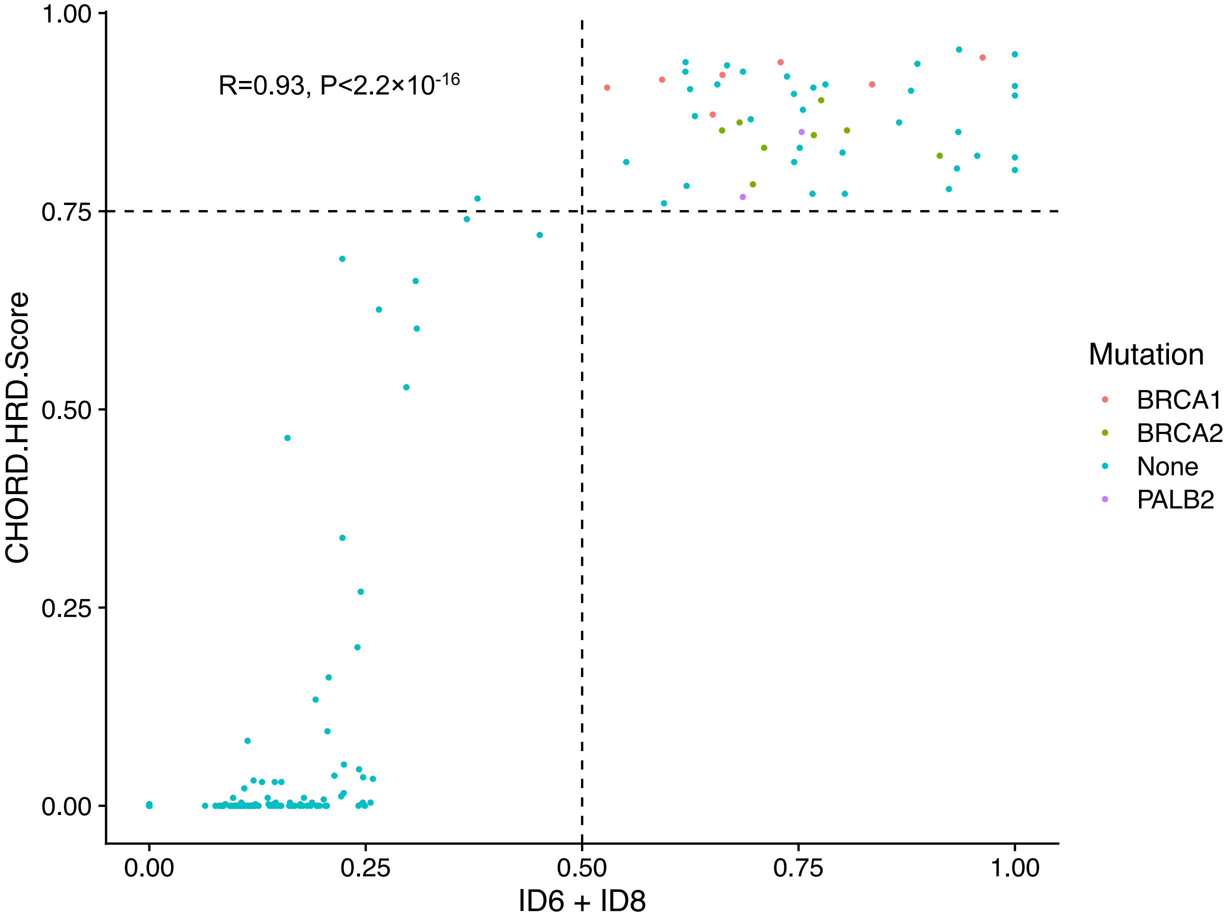

Supplementary Fig. 19 Correlation of CHORD-based HRD score with non-homologous end-joining (NHEJ) repair-associated INDEL signature activity (ID6+ID8). All tumors with mutations in homologous recombination-associated genes show NHEJ>0.5 and HRD>0.75 represented by vertical and horizontal dotted lines respectively. The color of each datapoint represents the mutated homologous recombination-associated gene. Pearson correlation coefficient (R) and two-sided unadjusted P value of its significance (*cor.test*) is shown. INDEL, indel and deletion.

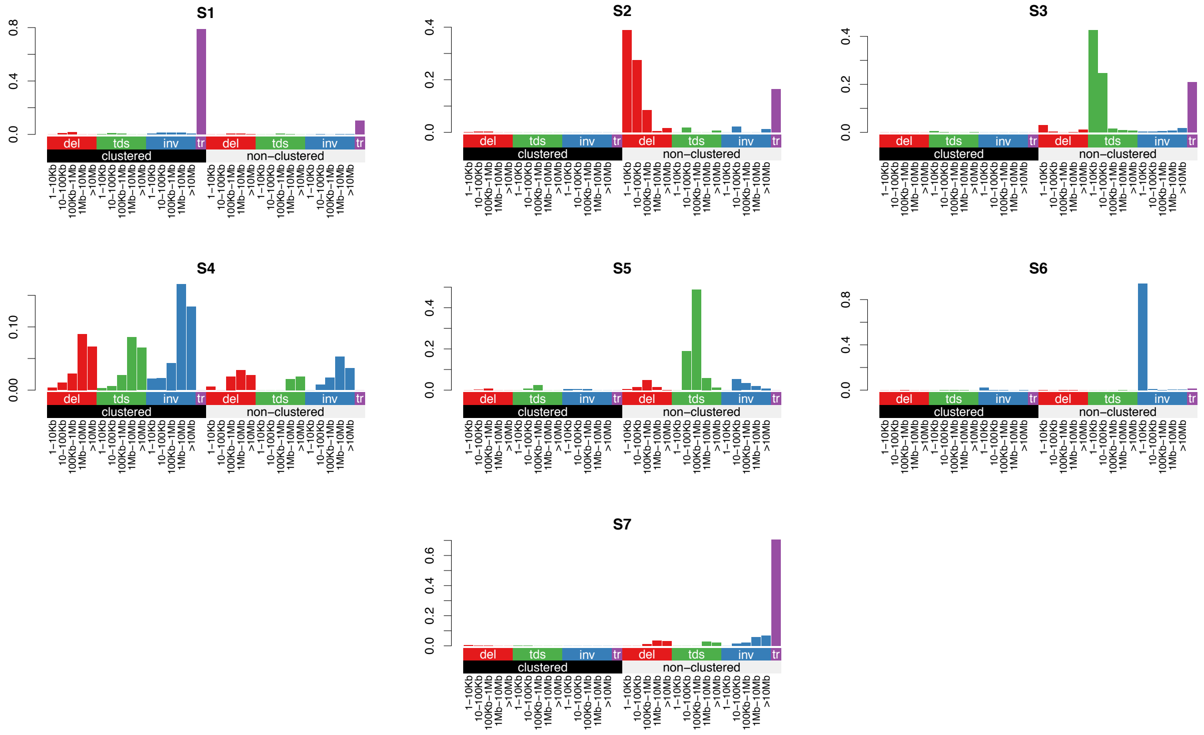

Supplementary Fig. 20 Seven structural variant signatures extracted using non-negative matrix factorization. Probability of rearrangement element on y-axis. Rearrangement elements are categorized by type and sorted by size within clustered and non-clustered groups on x-axis. del, deletion; tds, tandem duplication; inv, inversion; trans, translocation.

Cosine similarity
0.98984423

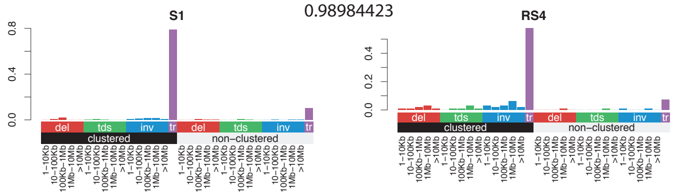

Cosine similarity
0.96125614

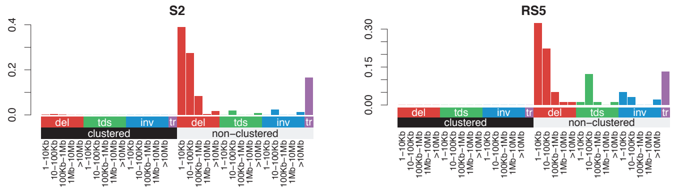

Cosine similarity
0.98301581

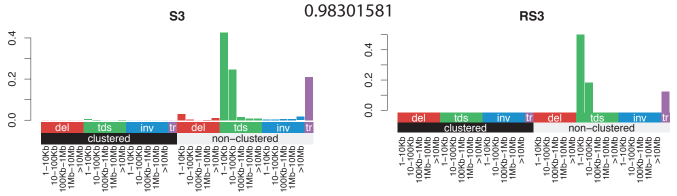

Cosine similarity
0.98223432

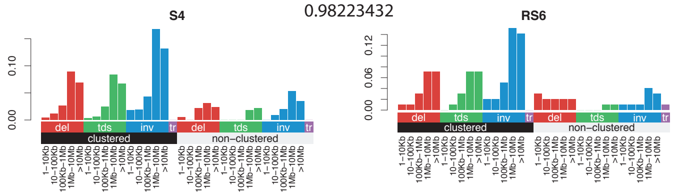

Cosine similarity
0.97587503

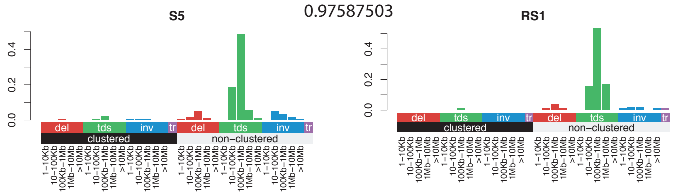

Cosine similarity
0.12722336

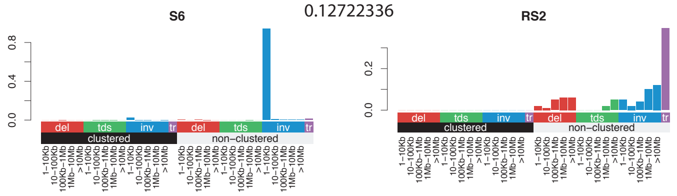

Cosine similarity
0.94182924

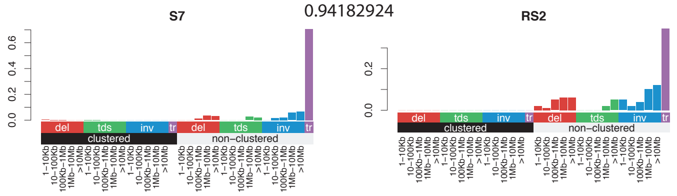

Supplementary Fig. 21 Comparison of the rearrangement signatures. Signature extracted from the current study is presented on the left while the most similar signature from Nik-Zainal *et al.* 2016 (PMID: 27135926) is presented on the right. Cosine similarity between each pair is displayed at the top of each rearrangement signatures pair plots. Probability of rearrangement element on y-axis. Rearrangement elements are categorized by type and sorted by size within clustered and non-clustered groups on x-axis. del, deletion; tds, tandem duplication; inv, inversion; trans, translocation.

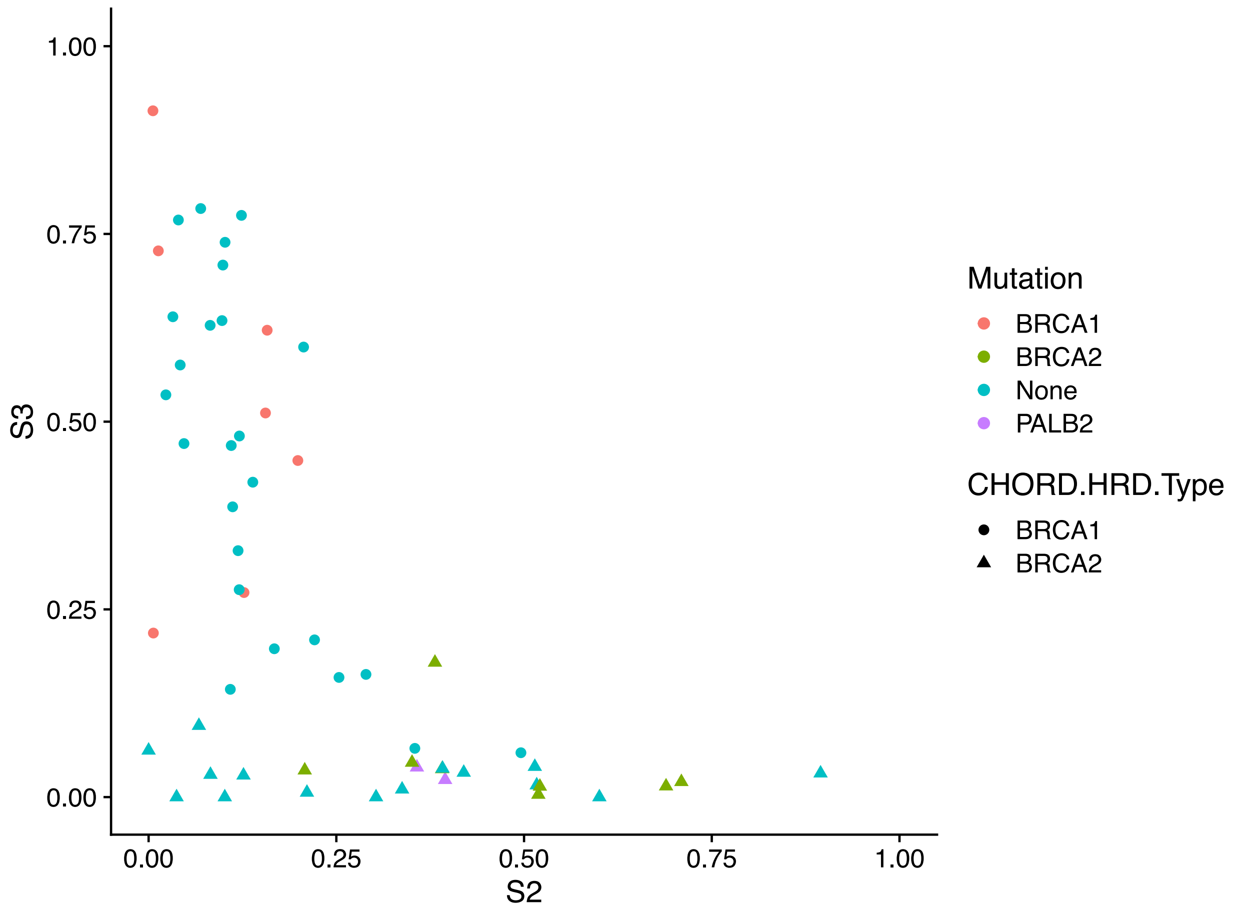

Supplementary Fig. 22 Activity of *BRCA*-associated SV (structural variant) signatures in CHORD-based HRD positive tumors. S2 and S3 are *BRCA2*- and *BRCA1*-associated SV signatures respectively. The color and shape of each datapoint represents the mutated homologous recombination-associated gene and the predicted CHORD-based HRD *BRCA*-type.

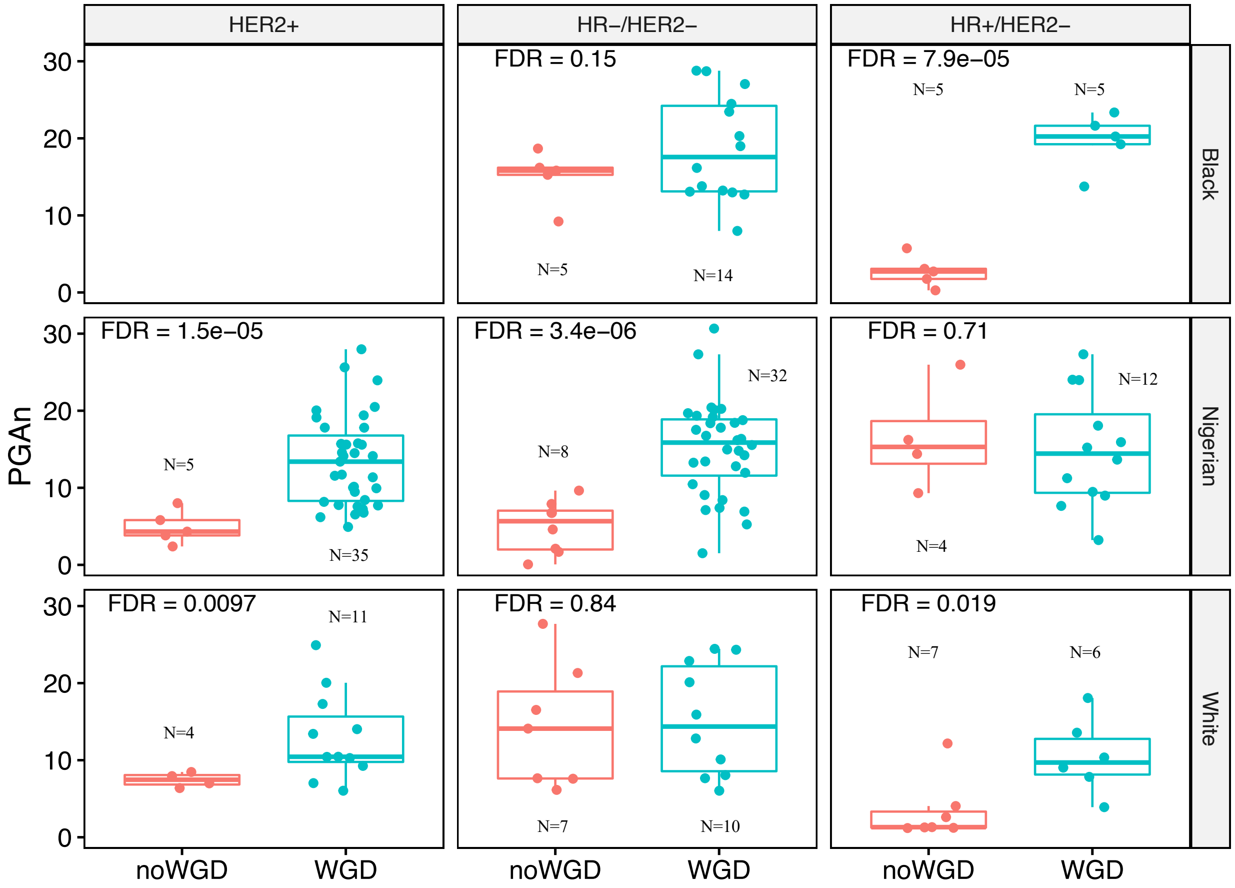

Supplementary Fig. 23 Comparison of PGA between WGD and non-WGD samples across groups and clinical subtypes. All Nigerian, Black and White samples are biologically independent samples. Sample size of each comparison group is indicated in the figure. Upper and lower whiskers extend to the largest and smallest values within (1.5 × interquartile range), respectively. T-test two-sided P values adjusted for multiple testing using FDR. PGA, the proportion of the genome altered; WGD, whole-genome duplication; HR, hormone receptor; HER2, human epidermal growth factor receptor 2.

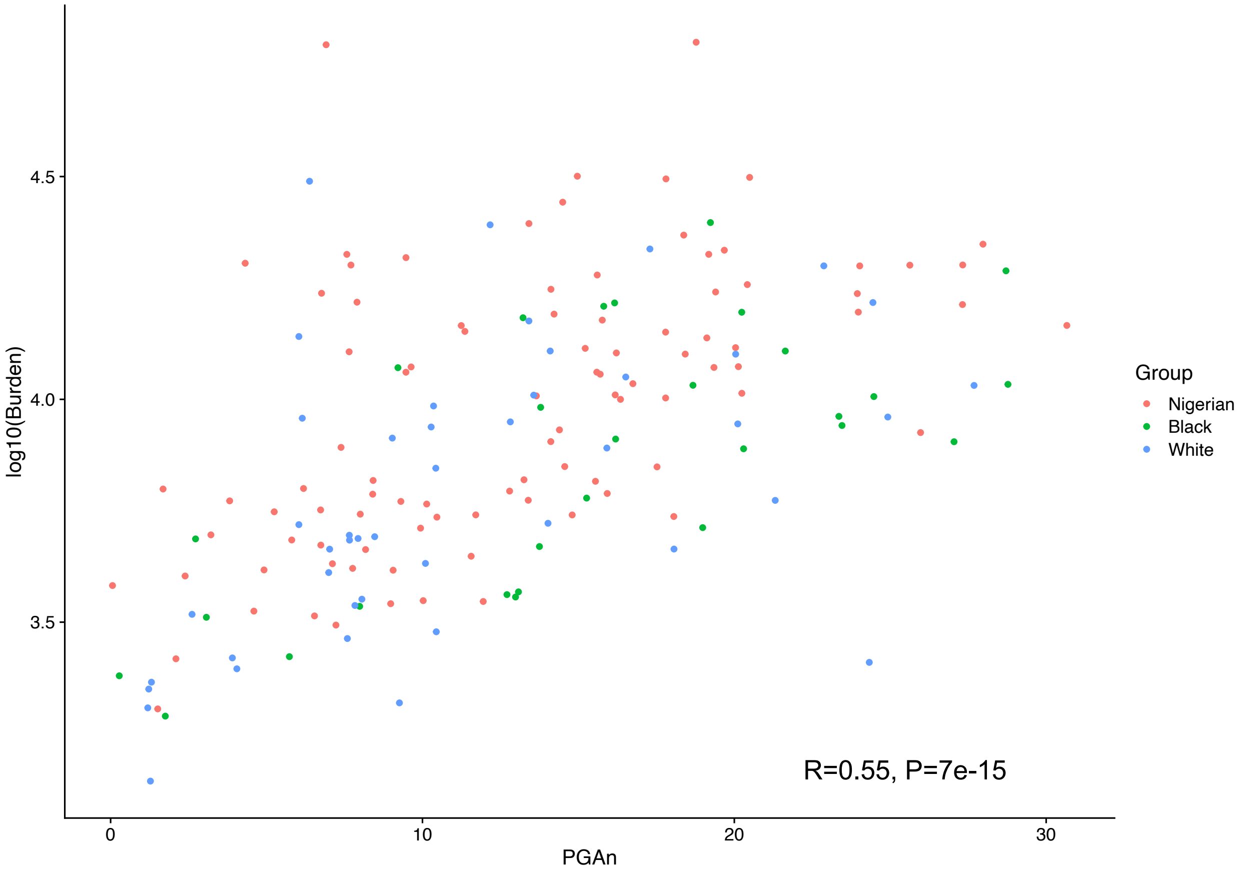

Supplementary Fig. 24 Correlation of PGAn and mutation burden. Pearson correlation coefficient (R) and two-sided unadjusted P value of its significance (*cor.test*) is shown. PGAn, the proportion of the genome altered adjusted with the number of copy number alteration segments.

N010842

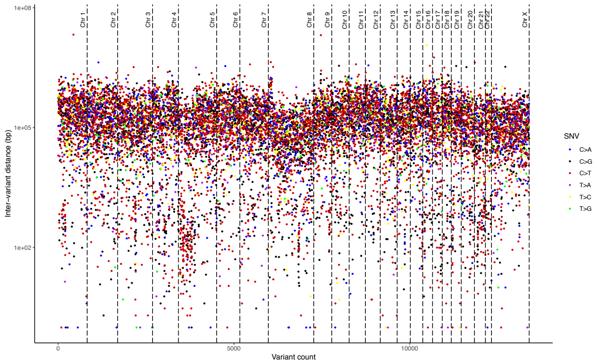

N010940

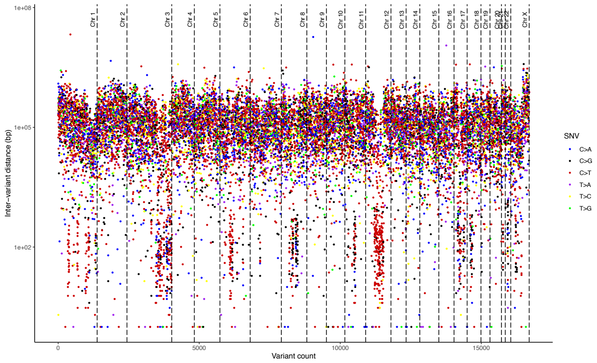

N011074

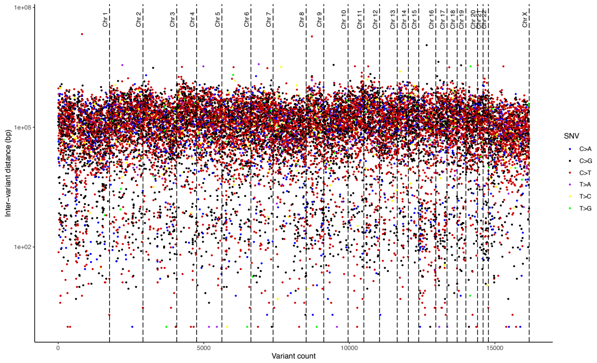

N011070

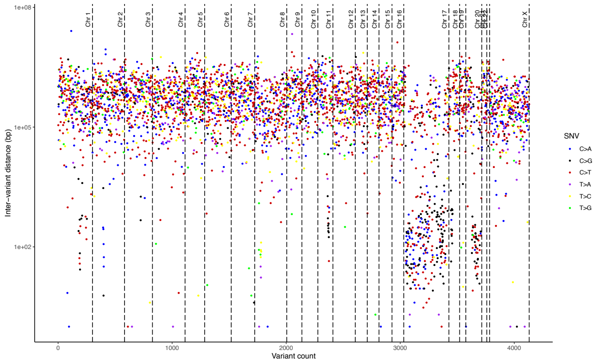

Supplementary Fig. 25 Kataegis profile of breast cancer tumors with high (n>10) foci rate. SNV, single nucleotide variant.

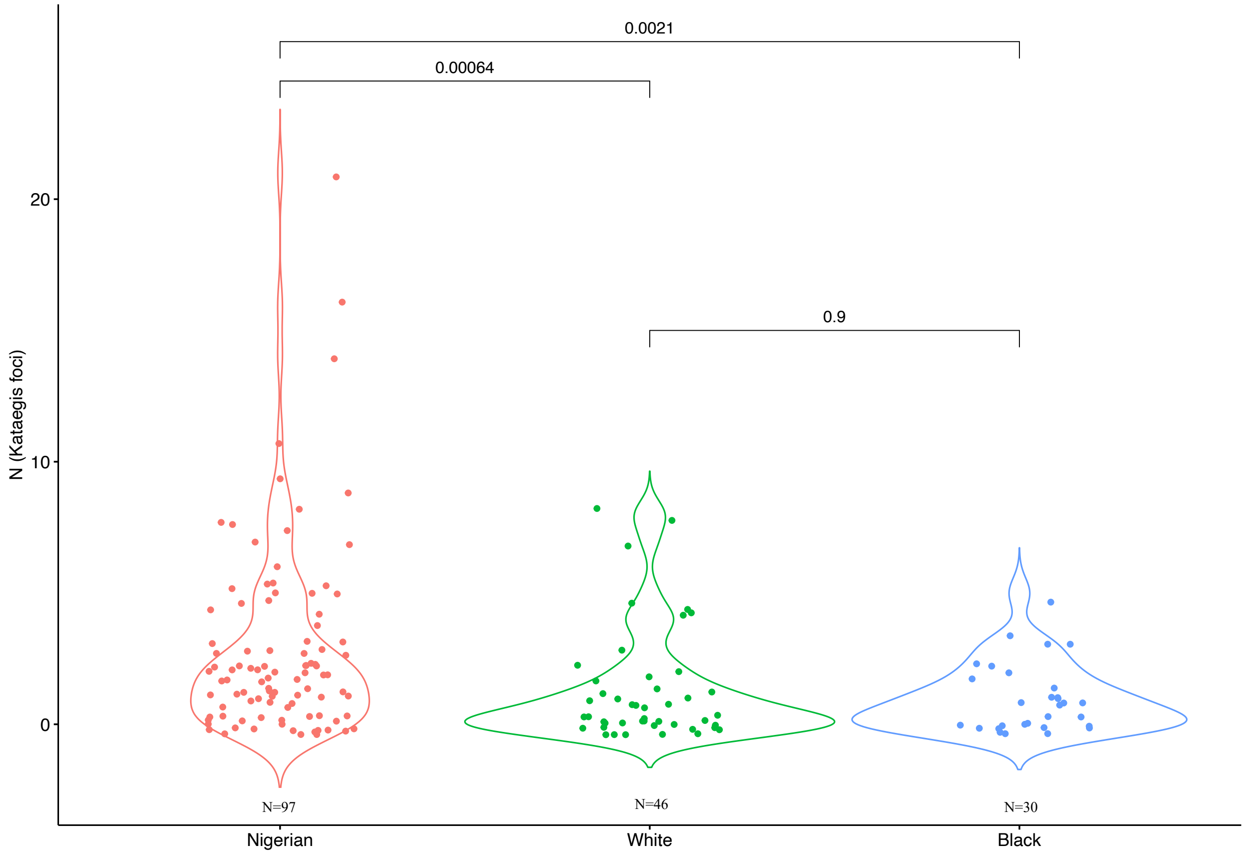

Supplementary Fig. 26 Comparison of kataegis foci rate across the three groups. All Nigerian, Black and White samples are biologically independent samples. Sample size of each comparison group is indicated in the figure. Two-sided Wilcoxon rank sum test unadjusted P values are shown.

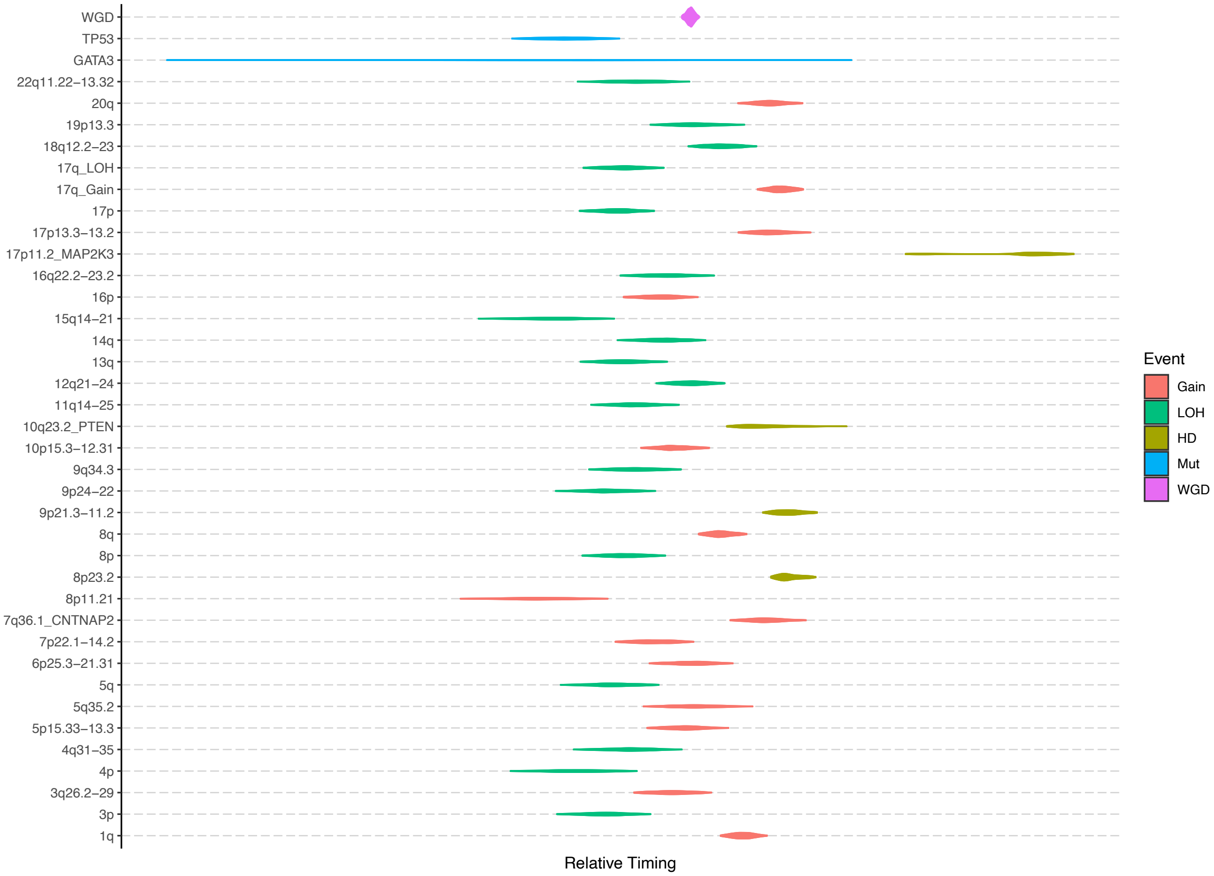

Supplementary Fig. 27 Chronological ordering of genomic events in White breast cancer tumors. Clonality-based ordering of significantly enriched copy number events (FDR<0.05), whole-genome duplication (WGD) and key frequent mutational drivers (*TP53* and *GATA3*) based on a Plackett-Luce model. LOH, loss of heterozygosity; HD, homozygous deletion; Mut, mutational driver.

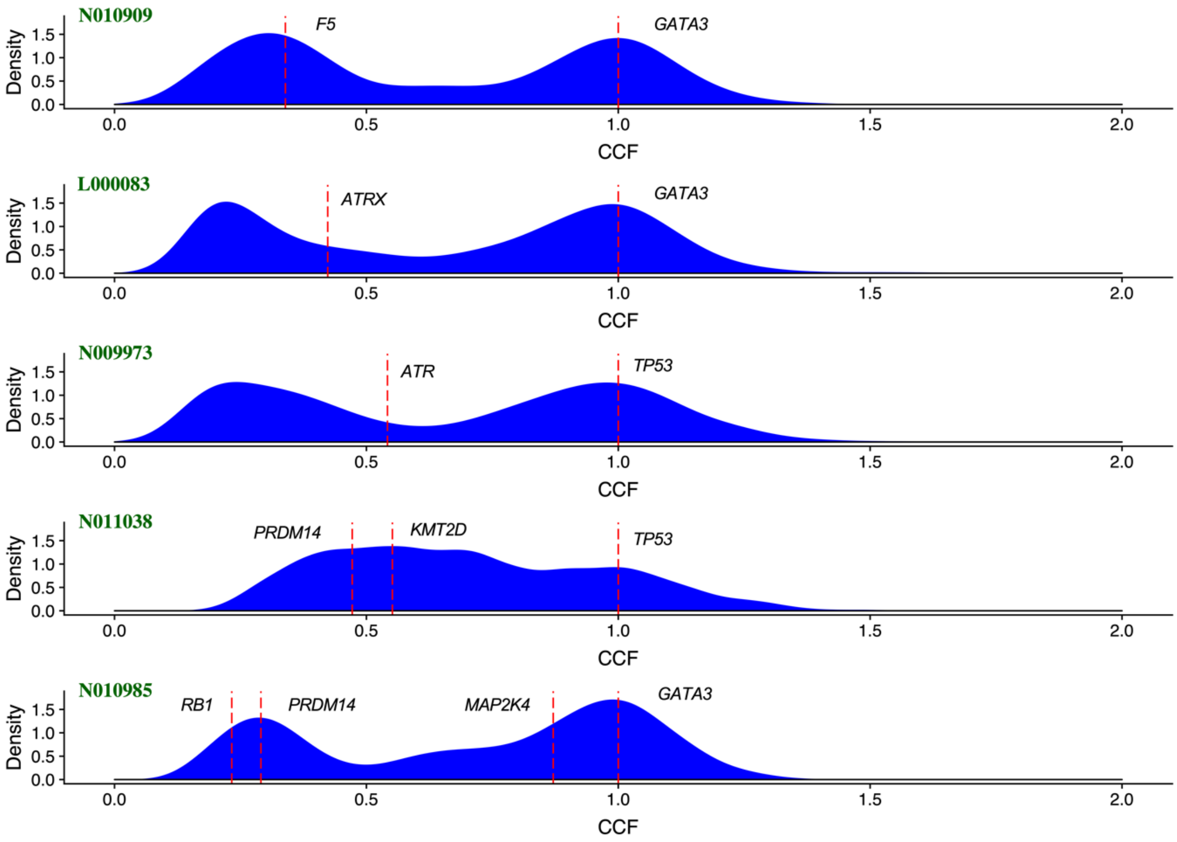

Supplementary Fig. 28 Cancer cell fraction (CCF) density distribution in tumors with the highest intra-tumoral heterogeneity. The CCF of most likely clonal and subclonal drivers in each tumor is shown.

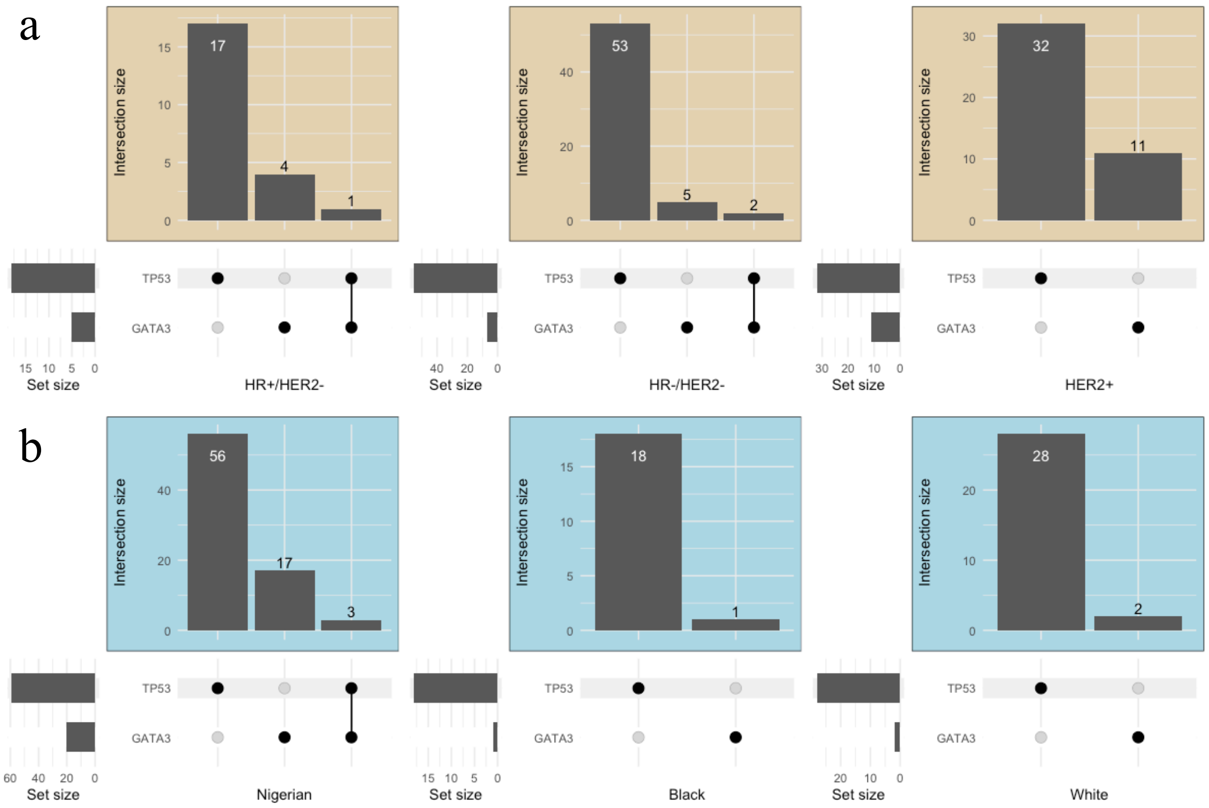

Supplementary Fig. 29 Upset plot of *TP53* and *GATA3* mutual exclusivity across **a** clinical subtypes and **b** ethnic groups analyzed in this study. Only 3 tumors (out of 173) harbored mutations in both genes. All Nigerian, Black and White samples are biologically independent samples. Sample size of each comparison group is indicated in the figure. HR, hormone receptor; HER2, human epidermal growth factor receptor 2.

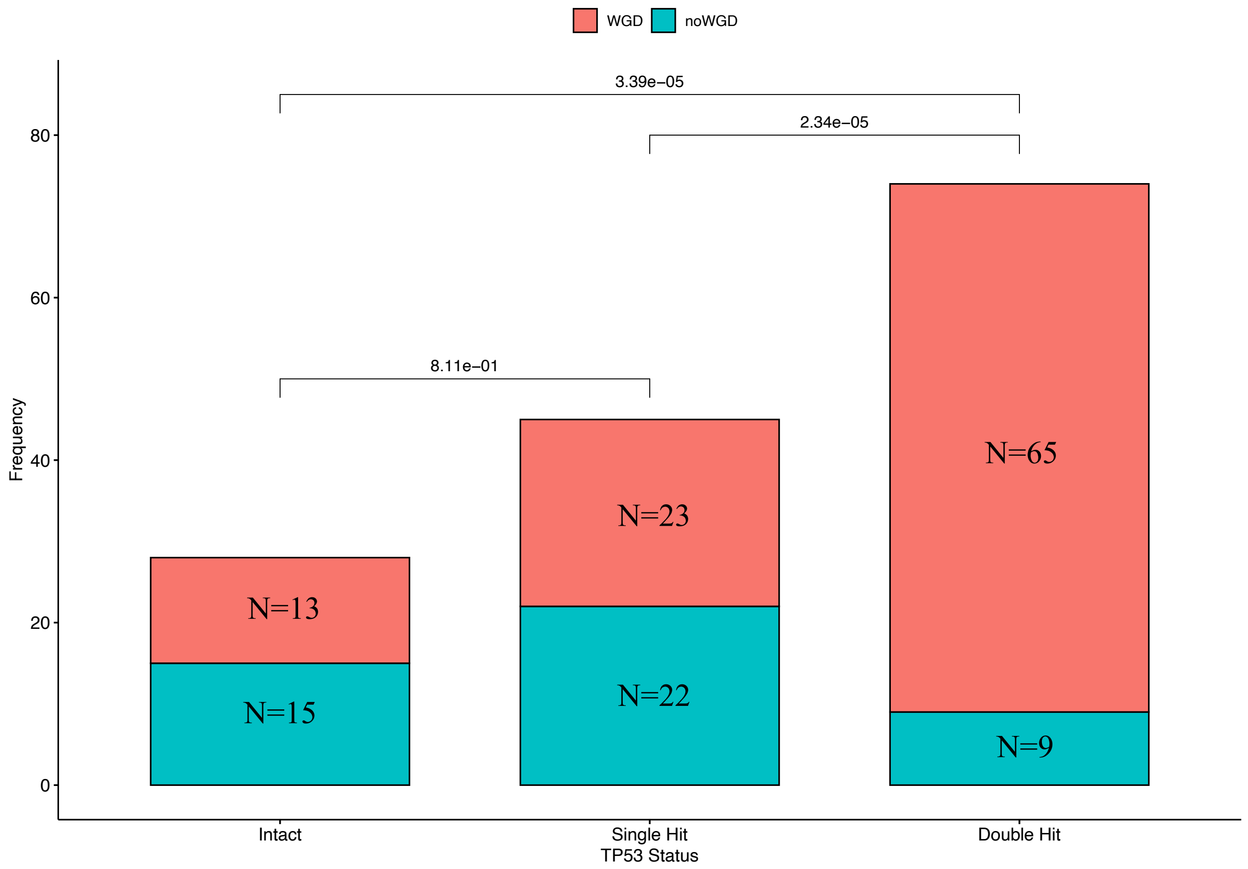

Supplementary Fig. 30: Association of *TP53* status with whole-genome duplication (WGD) rate. All samples are biologically independent samples. Sample size of each comparison group is indicated in the figure. Fisher exact test two-sided unadjusted P values are shown.

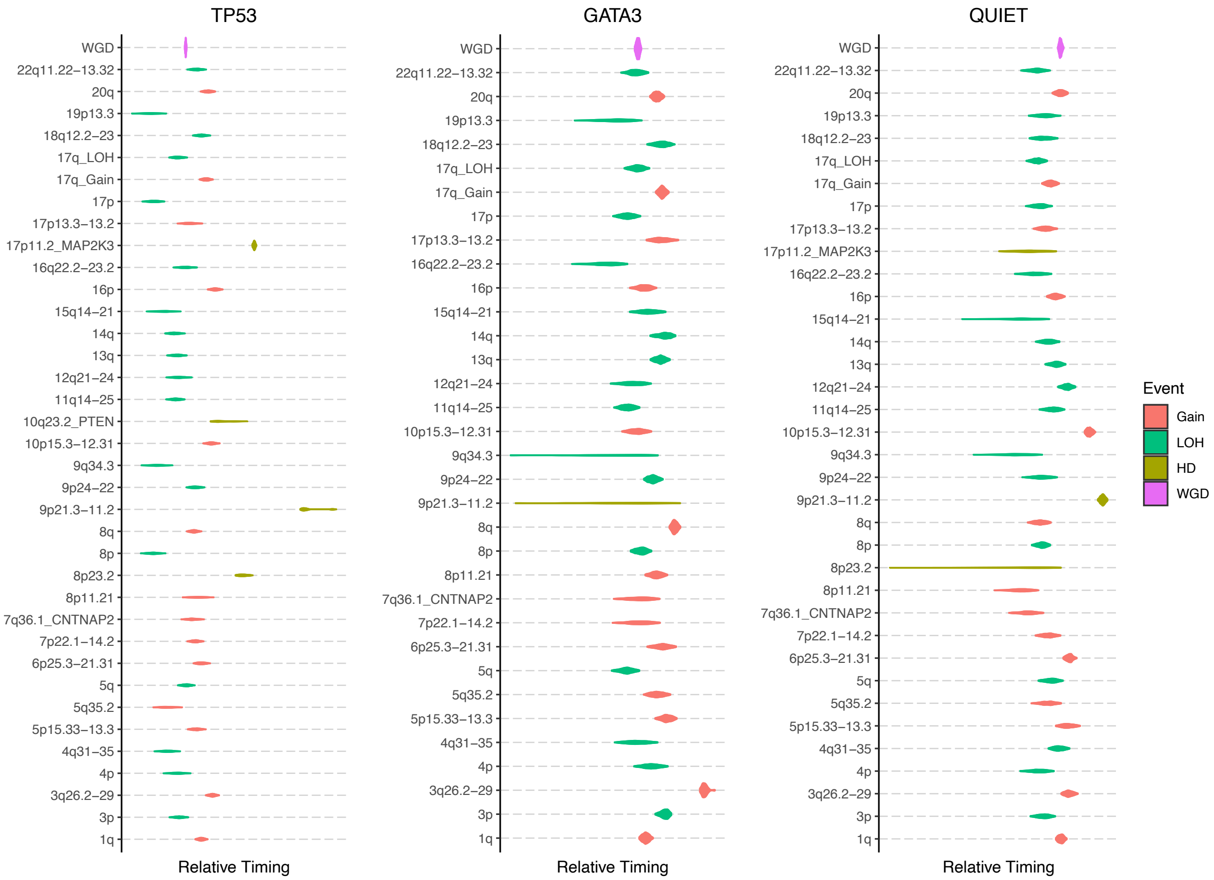

Supplementary Fig. 31 Chronological ordering of events within genomic subtypes of the Nigerian group. Clonality-based ordering of significantly enriched copy number events (FDR<0.05) and whole-genome duplication (WGD) based on a Plackett-Luce model. LOH, loss of heterozygosity; HD, homozygous deletion.

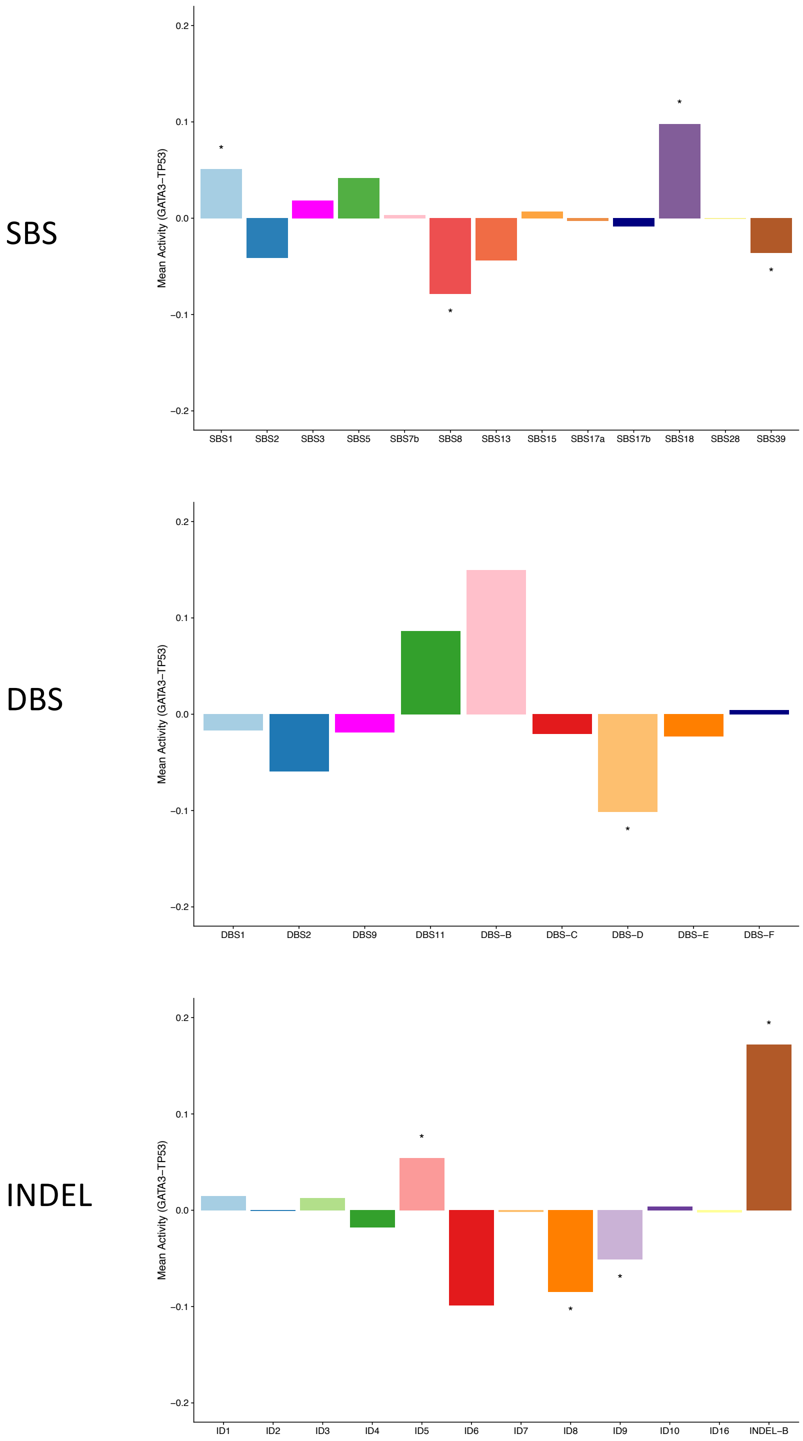

Supplementary Fig. 32 Differential activity of SBS, DBS and INDEL signatures between *GATA3* and *TP53* genomic subtypes. Asterisks represent significant differences after multiple testing correction (two-sided t-test P value adjusted using FDR; FDR<0.05). Signatures in the positive direction are more frequent in the *GATA3* subtype. SBS, single base substitution; DBS, double base substitution; INDEL, indel and deletion.

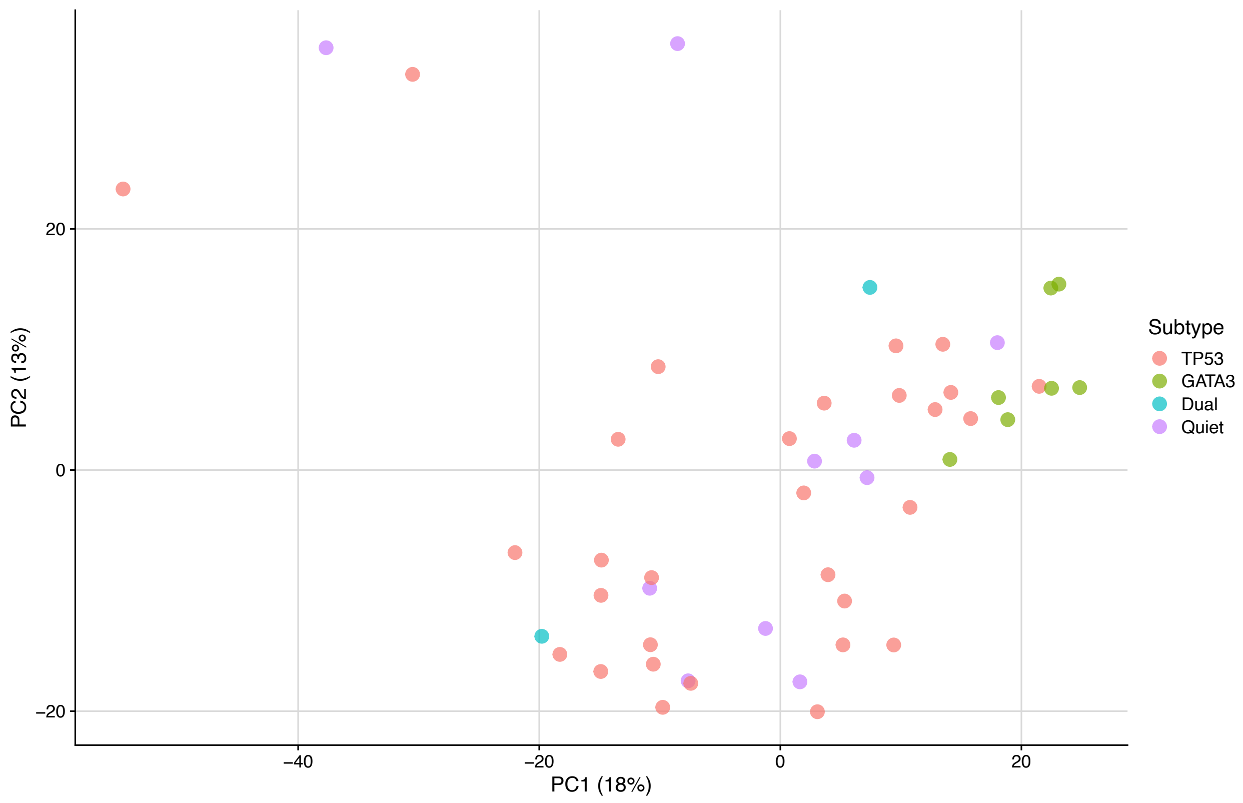

Supplementary Fig. 33 Principal components analysis of 49 Nigerian whole-transcriptomes representing the three genomic subtypes. Dual represents samples positive for both *GATA3* and *TP53*.

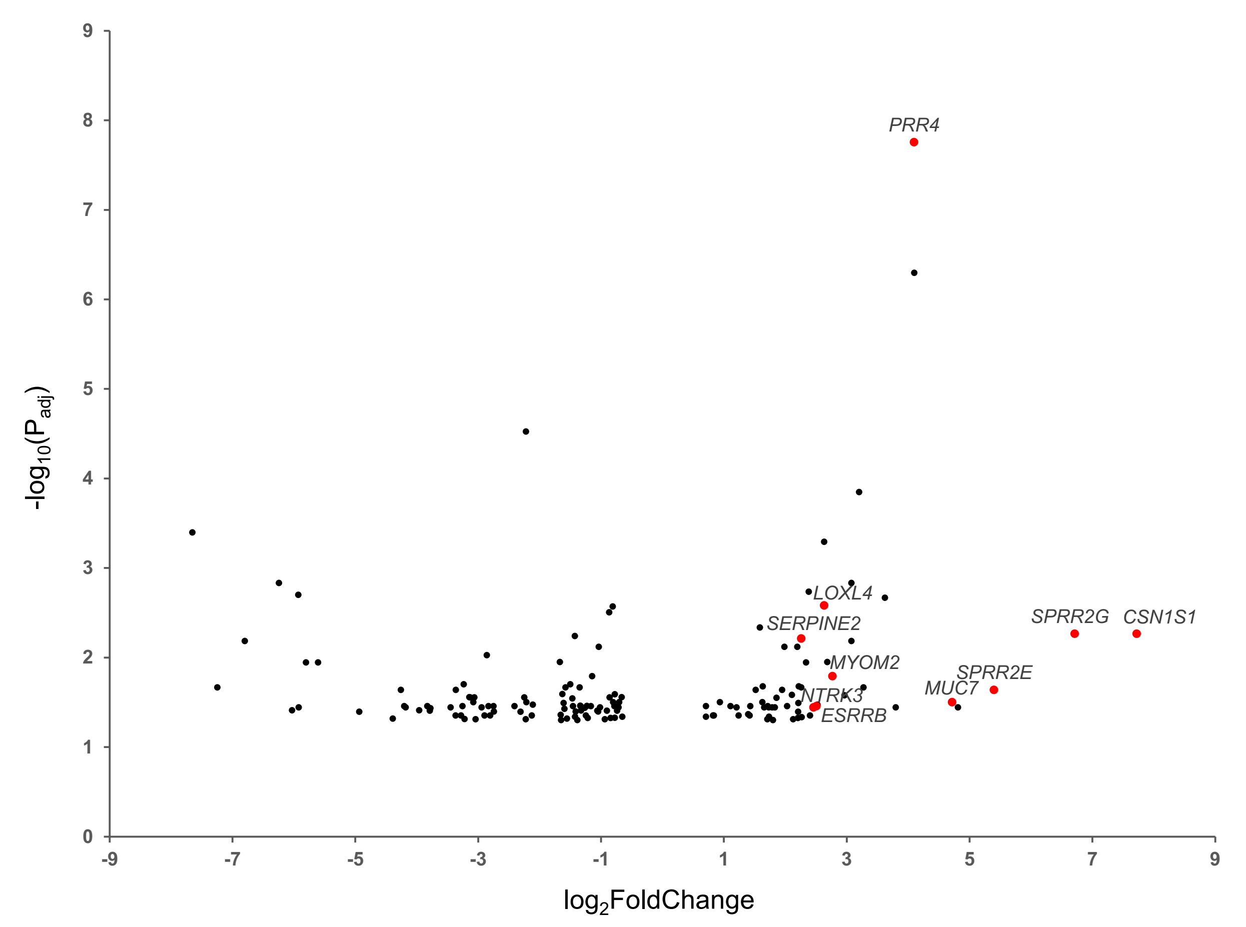

Supplementary Fig. 34: Volcano plot of differential expression analysis between quiet-genome tumors and *GATA3*/*TP53* positive tumors in the Nigerian group. Red dots demonstrate significant overexpression of genes previously associated with breast cancer. Differential expression analysis of raw read counts of protein-coding genes was performed using DESeq2. Significance was defined as false discovery rate (FDR)-adjusted two-sided P<0.05 and expression fold change >1.5.
