# Supplementary material for: Whole-genome analysis of Nigerian patients with breast cancer reveals ethnic-driven somatic evolution and distinct genomic subtypes
Source: Nat Commun. 2021 Nov 26;12:6946. doi: 10.1038/s41467-021-27079-w (PMC8626467; doi:10.1038/s41467-021-27079-w)
Supplement: Supplementary file 3 — Description of Additional Supplementary Files [file 41467_2021_27079_MOESM3_ESM.pdf]

Description of Additional Supplementary Files

File Name: Supplementary Data 1

Description: Details of germline and somatic *BRCA*-positive patients.

File Name: Supplementary Data 2

Description: Number of kataegis foci in all breast cancer tumors.
