# Supplementary material for: Whole-genome analysis of Nigerian patients with breast cancer reveals ethnic-driven somatic evolution and distinct genomic subtypes
Source: Nat Commun. 2021 Nov 26;12:6946. doi: 10.1038/s41467-021-27079-w (PMC8626467; doi:10.1038/s41467-021-27079-w)
Supplement: Supplementary file 6 — Reporting Summary [file 41467_2021_27079_MOESM6_ESM.pdf]

Corresponding author(s): David C. Wedge, Olufunmilayo I. Olopade

Last updated by author(s): October 28, 2021

Reporting Summary

Nature Research wishes to improve the reproducibility of the work that we publish. This form provides structure for consistency and transparency in reporting. For further information on Nature Research policies, see our Editorial Policies and the Editorial Policy Checklist.

Statistics

For all statistical analyses, confirm that the following items are present in the figure legend, table legend, main text, or Methods section.

n/a	Confirmed	
☐	☒	The exact sample size (*n*) for each experimental group/condition, given as a discrete number and unit of measurement
☐	☒	A statement on whether measurements were taken from distinct samples or whether the same sample was measured repeatedly
☐	☒	The statistical test(s) used AND whether they are one- or two-sided<br>*Only common tests should be described solely by name; describe more complex techniques in the Methods section.*
☐	☒	A description of all covariates tested
☐	☒	A description of any assumptions or corrections, such as tests of normality and adjustment for multiple comparisons
☐	☒	A full description of the statistical parameters including central tendency (e.g. means) or other basic estimates (e.g. regression coefficient) AND variation (e.g. standard deviation) or associated estimates of uncertainty (e.g. confidence intervals)
☐	☒	For null hypothesis testing, the test statistic (e.g. *F*, *t*, *r*) with confidence intervals, effect sizes, degrees of freedom and *P* value noted<br>*Give P values as exact values whenever suitable.*
☐	☒	For Bayesian analysis, information on the choice of priors and Markov chain Monte Carlo settings
☒	☐	For hierarchical and complex designs, identification of the appropriate level for tests and full reporting of outcomes
☐	☒	Estimates of effect sizes (e.g. Cohen's *d*, Pearson's *r*), indicating how they were calculated

Our web collection on statistics for biologists contains articles on many of the points above.

Software and code

Policy information about availability of computer code

Data collection	No specific code was used in the data collection process.
Data analysis	BWA-MEM (v0.7.12; http://bio-bwa.sourceforge.net/)<br>PicardTools MarkDuplicates (v1.119; https://broadinstitute.github.io/picard/)<br>Platypus (v0.7.9.1; https://github.com/andyrimmer/Platypus)<br>MuTect (v1.1.7; https://software.broadinstitute.org/cancer/cga/mutect)<br>Strelka (v1.0.13; ftp://strelka:@ftp.illumina.com/v1-branch/v1.0.13/)<br>cgpPindel (v3.0.1) within cgpWGS container (v2.0.1; https://dockstore.org/containers/quay.io/wtsicgp/dockstore-cgpwgs:2.0.1?tab=info)<br>ANNOVAR (version May2018; http://annovar.openbioinformatics.org/)<br>vafCorrect (v5.7.0; https://github.com/cancerit/vafCorrect)<br>cDriver (v0.4.2; https://github.com/hanasusak/cDriver)<br>MutSigCV (v1.3; https://software.broadinstitute.org/cancer/cga/mutsig)<br>Maftools (v2.6.05; https://www.bioconductor.org/packages/release/bioc/html/maftools.html)<br>SigProfilerExtractor (v0.0.5.77; https://github.com/AlexandrovLab/SigProfilerExtractor)<br>Manta (v1.1.0; https://github.com/Illumina/manta)<br>DELLY (v0.7.0; https://github.com/dellytools/delly)<br>Lumpy (v0.2.13; https://github.com/arq5x/lumpy-sv)<br>SURVIVOR (v1.0.6; https://github.com/fritzsedlazeck/SURVIVOR)<br>CHORD (v2.0; https://github.com/UMCUGenetics/CHORD)<br>Battenberg (v2.2.8; https://github.com/Wedge-lab/battenberg)<br>KataegisPCF (v1.0; https://github.com/nansari-pour/KataegisPCF)<br>PlackettLuce package in R (v0.3.0; https://github.com/hturner/PlackettLuce)

DPClust (v2.2.2; https://github.com/Wedge-lab/dpclust)
STAR (v2.4.2a; https://github.com/alexdobin/STAR)
HTSeq (v0.6.1p1; https://github.com/htseq/htseq)
RNA-SeQC (v1.1.8; https://software.broadinstitute.org/cancer/cga/rna-seqc)
featureCounts (v1.5.1; http://subread.sourceforge.net/)
PicardTools (v1.128; https://broadinstitute.github.io/picard/)
SAMtools (v1.3.1; http://www.htslib.org/)
DESeq2 (v1.24.0; https://bioconductor.org/packages/release/bioc/html/DESeq2.html)

For manuscripts utilizing custom algorithms or software that are central to the research but not yet described in published literature, software must be made available to editors and reviewers. We strongly encourage code deposition in a community repository (e.g. GitHub). See the Nature Research guidelines for submitting code & software for further information.

Data

Policy information about availability of data

All manuscripts must include a data availability statement. This statement should provide the following information, where applicable:

- Accession codes, unique identifiers, or web links for publicly available datasets
- A list of figures that have associated raw data
- A description of any restrictions on data availability

The raw sequencing data and the processed genomic data from Nigerian cases have been deposited in dbGaP under Study Accession phs001687.v1.p1 (https://www.ncbi.nlm.nih.gov/projects/gap/cgi-bin/study.cgi?study_id=phs001687.v1.p1). TCGA raw sequencing data are available in dbGaP under Study Accession phs000178.v11.p8 (https://www.ncbi.nlm.nih.gov/projects/gap/cgi-bin/study.cgi?study_id=phs000178.v11.p8). Data access to dbGaP can be obtained by contacting National Cancer Institute Data Access Committee (NCIDAC@mail.nih.gov). Access to TCGA variant calls that support the findings of this study are available on request to the corresponding author (O.I.O) from the requestor who has approved authorized access to TCGA controlled data. The remaining data are available within the Article and Supplementary Information. Source data are provided with this paper.

Field-specific reporting

Please select the one below that is the best fit for your research. If you are not sure, read the appropriate sections before making your selection.

☒ Life sciences ☐ Behavioural & social sciences ☐ Ecological, evolutionary & environmental sciences

For a reference copy of the document with all sections, see nature.com/documents/nr-reporting-summary-flat.pdf

Life sciences study design

All studies must disclose on these points even when the disclosure is negative.

Sample size	No sample-size calculation was performed. 76 raw breast cancer whole-genome sequencing (WGS) data were downloaded directly from The Cancer Genome Atlas (TCGA) Data Portal, and all Nigerian WGS passed QC (n=97, 46 of them also had RNA-seq data available) were used for this analysis. Larger datasets would have given us more power in identifying differential signals; however, with the White TCGA and Nigerian cohorts analyzed, we were still able to detect GATA3 and ZNF217/SYPL1 enrichment in the Nigerians. The TCGA cohort was used for comparative analysis mainly due to data accessibility (availability of raw FASTQs) because we wanted to process all Nigerian and non-Nigerian samples using a uniform pipeline. There is paucity of African breast cancer genomes and this study will now provide the largest dataset of African breast cancer genomes for future comparative analysis.
Data exclusions	High-depth WGS was performed on 100 breast tumors (90X depth) and paired normal tissue (30X depth) from women with breast cancer in Southwest Nigeria. Three samples were excluded due to low purity estimates (<10%).
Replication	DNA sequencing data from all Nigerian and TCGA samples was uniformly processed. Data consistency was tested and confirmed by comparing the variant calls of WGS and WES data where both were available from the same patient (Pitt, J.J. et al. Nat Commun, 2018; PMID: 30327465).
Randomization	Samples were allocated to experimental groups based on their race/ethnicity after estimating the genetic ancestry of all TCGA breast cancer patients using principal component analysis. All Nigerian patients were assumed to be 100% African with little to no admixture with other populations. Data were further partitioned into cancer sub-type based on immunohistochemistry.
Blinding	Blinding was not relevant to analysis because our study is a comparative analysis across different groups/sub-groups according to ethnicity, race, and cancer subtypes.

Reporting for specific materials, systems and methods

We require information from authors about some types of materials, experimental systems and methods used in many studies. Here, indicate whether each material, system or method listed is relevant to your study. If you are not sure if a list item applies to your research, read the appropriate section before selecting a response.

Materials & experimental systems

n/a	Involved in the study
☐	☒ Antibodies
☒	☐ Eukaryotic cell lines
☒	☐ Palaeontology and archaeology
☒	☐ Animals and other organisms
☐	☒ Human research participants
☒	☐ Clinical data
☒	☐ Dual use research of concern

Methods

n/a	Involved in the study
☒	☐ ChIP-seq
☒	☐ Flow cytometry
☒	☐ MRI-based neuroimaging

Antibodies

Antibodies used	Commercial antibodies used:<br>ER -- rabbit monoclonal antibody, clone SP1 (Thermo Scientific, Cat# RM-9101); 1:100 dilution<br>PR -- rabbit monoclonal antibody, clone SP2 (Thermo Scientific, Cat# RM-9102); 1:100 dilution<br>HER2 -- rabbit anti-human antibody, HercepTest Kit (Dako, Cat# K520421-5); no dilution/ready to use
Validation	The validation statement of commercial antibodies are available from the manufactures:<br>ER -- https://tools.thermofisher.com/content/sfs/brochures/D12524~.pdf<br>PR -- https://tools.thermofisher.com/content/sfs/brochures/D12527~.pdf<br>HER2 -- https://www.agilent.com/store/en_US/Prod-K520421-5/K520421-5; https://www.agilent.com/cs/library/usermanuals/public/28630_herceptest_interpretation_manual-breast_ihc_row.pdf

Human research participants

Policy information about studies involving human research participants

Population characteristics	The charachteristics of the participants within this study is outlined in the Supplementary Tables.
Recruitment	A grand total of 493 subjects were recruited from University College Hospital, Ibadan (UCH; N=284) and Lagos State University Teaching Hospital (LASUTH; N=209) between February 2013 and September 2015. Cases were recruited at first presentation, and all patients were female. No control population cohort were recruited for this study. Six biopsy cores and peripheral blood were collected from each patient. Two biopsy cores were used for routine formalin fixation for clinical diagnosis and the remaining four cores were preserved in PAXgene Tissue containers (Qiagen, CA) for subsequent genomic material extraction. In addition, 27 mastectomy tissues were preserved in RNAlater. Complete pathology assessment was performed centrally by study pathologists. Tumor quality was assessed based on cellularity, histology type, and morphological quality of tissue using TCGA best practices.<br>In the manuscript we stated the limitation that both Nigerian Breast Cancer Study (NBCS) and TCGA are conducted on convenient and purposive samples ascertained in hospitals and may not reflect the origin populations. In addition, HER2+ subtype is unavailable in WGS samples in TCGA Black group. Adjusting for subtype is important for proper comparison. We therefore had taken into account subtype and corrected for this in comparisons.
Ethics oversight	This study was embedded within the Nigerian Breast Cancer Study (NBCS) and approved by the Institutional Review Board of all participating institutions: The University of Chicago, University College Hospital, Ibadan (UCH), and Lagos State University Teaching Hospital (LASUTH). Each patient gave written informed consent before participation in the study.

Note that full information on the approval of the study protocol must also be provided in the manuscript.
